# Efficient Electron Transfer Driven by Excited-State Structural Relaxation in Corrole–Perylenedimiide Dyad

**DOI:** 10.1021/acs.jpclett.4c00916

**Published:** 2024-05-08

**Authors:** Damian Kusy, Hongwei Song, Antoni Rząca, Marzena Banasiewicz, Cristina A. Barboza, Dongho Kim, Daniel T. Gryko

**Affiliations:** 1 Institute of Organic Chemistry, Polish Academy of Sciences, Kasprzaka 44/52, 01-224 Warsaw, Poland; 2 Spectroscopy Laboratory for Functional π-Electronic Systems and Department of Chemistry, Yonsei University, Seoul 03722, Republic of Korea; 3 Faculty of Chemistry, Warsaw University, Pasteura 1, 02-093 Warsaw, Poland; 4 Institute of Physics, Polish Academy of Sciences, Al. Lotników 32/46, 02-668 Warsaw, Poland; 5 Institute of Advanced Materials, Faculty of Chemistry, Wrocław University of Science and Technology, Wybrzeże Wyspiańskiego 27, 50-370 Wrocław, Poland

## Abstract

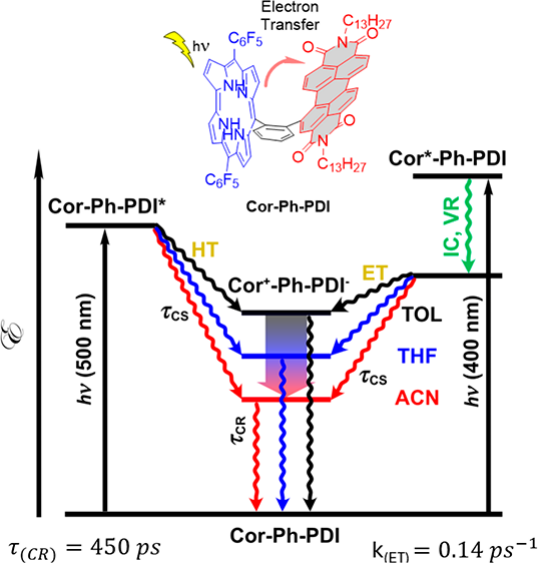

A sterically encumbered *trans*-A_2_B-corrole possessing a perylenediimide (PDI) scaffold in close proximity to the macrocycle has been synthesized via a straightforward route. Electronic communication as probed via steady-state absorption or cyclic voltammetry is weak in the ground state, in spite of the corrole ring and PDI being bridged by an *o*-phenylene unit. The TDDFT excited-state geometry optimization suggests after excitation the interchromophoric distance is markedly reduced, thus enhancing the through-space electronic coupling between the corrole and the PDI. This is corroborated by the strong deviation of the emission spectrum originating from both PDI and corrole in the dyad. Selective excitation of both donor and acceptor units triggers efficient sub-picosecond electron transfer and hole transfer, respectively, followed by fast charge recombination. In comparison to previously studied corrole–PDI dyads, both charge separation and charge recombination occur faster, because of the structural relaxation in the excited state.

Various vital processes, including photosynthesis^1,2^ and DNA repair,^3,4^ hinge upon the intricate phenomenon of electron transfer (ET).^5^ The intricacies inherent in investigating ET within natural photosynthetic systems have prompted the utilization of model systems comprising donor and acceptor units to attain comprehensive insights. Despite considerable strides facilitated by the study of diverse model systems,^6,7^ substantial challenges persist.^8−10^ The endeavor to engineer artificial light-harvesting systems has spurred the development of arrays incorporating synthetic chromophores,^11−15^ effectively mimicking pivotal steps in photosynthesis.^16−25^ Amidst various structural scaffolds meeting essential photophysical prerequisites, our research, along with others, has demonstrated the applicability of free-base corroles^26−29^ as chromophores in constructing bichromophoric systems adept at energy or electron transfer processes.^30−39^

Our prior investigations have established that corrole and perylenediimides (PDIs)^40,41^ exhibit thermodynamic compatibility, fostering electron transfer within weakly coupled systems.^42−46^ Specifically, an efficient electron transfer from corrole, serving as an electron donor, to PDI, acting as an electron acceptor, is observed when the chromophores are tethered by a medium-length amide-based bridge^42−44^ or when a short peptide functions as the bridge.^45^

The objective of this study is to strategically amalgamate corrole and PDI in a geometric configuration where both chromophores are in close proximity, while simultaneously demonstrating weak electronic coupling through bonds. We propose that this architectural arrangement will impart the desired photophysical properties to the resulting dyads, characterized by weak coupling in the ground state and highly efficient electron transfer reactions in the excited state. Computational studies for the newly synthesized Cor-Ph-PDI indicate that, in the ground state, the two chromophores are in roughly perpendicular orientation, resulting in an extended donor–acceptor distance and significant angle between the centers of mass of each macrocycle. However, in the excited state, interchromophoric torsional motion shortens the donor–acceptor distance via reduction of the angle between the two π-systems. This structural relaxation facilitates through-space electronic communication between the corrole ring and PDI in the excited state, leading to remarkably efficient sub-picosecond electron/hole transfer.

The design of a new corrole–perylenediimide dyad is based on the following principle: bridging the donor (corrole) and acceptor (PDI) via benzene ring securing, in principle, electronic communication while simultaneously distorting the molecule so that an acceptor is situated “above the donor” (through-space proximity). Attempting to take advantage of well-developed synthesis of *trans*-A_2_B-corroles^47,48^ we resolved to place a PDI unit at position 10 of a corrole macrocycle bridged together via *o*-phenylene unit. The two electron-withdrawing C_6_F_5_ groups at positions 5 and 15 are critical to the structure, as they serve to stabilize the corrole core.^49,50^ To obtain the target compounds, we began with the synthesis of the appropriate aldehyde PDI-PhCHO (Supporting Information Scheme S1). The initial step involved bromination of diimide PDI^51−54^ derived from perylene-3,4,9,10-tetracarboxylic dianhydride to achieve intermediate PDI-Br. Subsequently, the desired aldehyde PDI-PhCHO was prepared via the Suzuki–Miyaura reaction in 97% yield. Finally, PDI-PhCHO and 5-(pentafluorophenyl)dipyrrane^55,56^ were subjected to conditions optimized for the synthesis of *trans*-A_2_B-corrole from electron-deficient dipyrranes,^57^ which led to the formation of dyad Cor-Ph-PDI in 18% yield (Scheme S1 and Figure 1). The required model Cor-BiPh was synthesized analogously from [1,1′-biphenyl]-2-carbaldehyde in a 14% yield (Scheme S2 and Figure 1).

**Figure 1:**
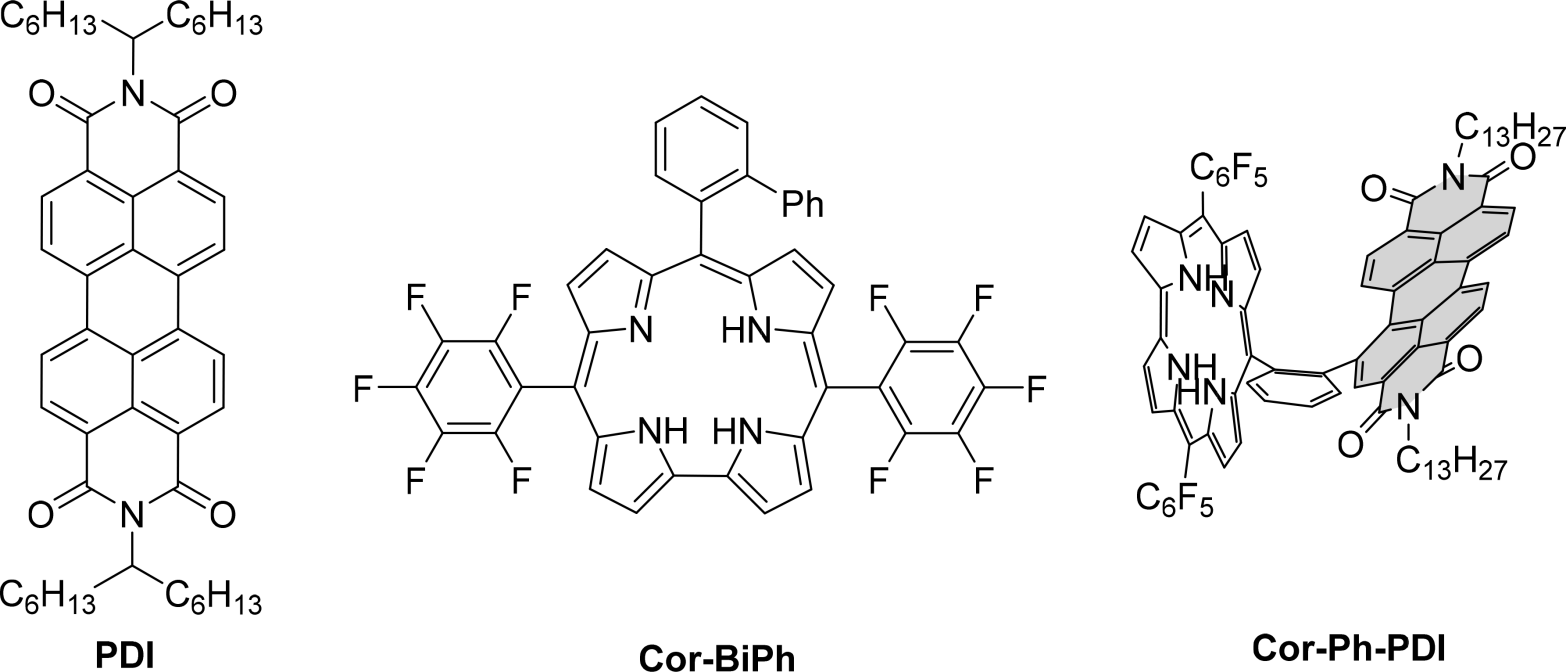
Structures of the studied dyes.

During the design of the final compound molecule Cor-Ph-PDI, we utilized a branched imide substituent CH(C_6_H_13_)_2_ (swallow-tail substituents), which not only enhances solubility but also prevents aggregation at high concentrations.^58^

Corroles are recognized for exhibiting irreversible oxidation at potentials that vary depending on the arrangement of substituents.^59^ Electrochemical investigations were conducted on PDI, Cor-BiPh, and Cor-Ph-PDI (Figure 1) to determine the degree of electronic coupling in the dyad. Examples of cyclic voltammograms illustrating the reduction and oxidation processes of Cor-BiPh and Cor-Ph-PDI are presented in Figures S6 and S7. A summary of the redox potentials is provided in Table 1.

**Table 1:** Redox Potentials of Cor-BiPh and Cor-Ph-PDI in Dichloromethane^a^

Compound	*E*_ox_^PA^ (V)	*E*_ox_^PC^ (V)	*E*_ox_^1/2^ (V)	*E*_ox_^onset^ (V)	IP^b^ (eV)	*E*_red_^PA^ (V)	*E*_red_^PC^ (V)	*E*_red_^1/2^ (V)	*E*_red_^onset^ (V)	EA^c^ (eV)	*E*_gap_ (eV)
Cor-Ph-PDI	0.87			0.70	–5.04	–0.56	–0.78	–0.67	–0.53	–3.81	1.23
	1.02	0.86	0.94			–0.80	–1.01	–0.90			
	1.21										
Cor-BiPh	0.80			0.60	–4.94	–0.70	–0.95	–0.83	–0.51	–3.83	1.11
	0.96										
	1.04	0.92	0.98								
PDI								–0.58^60^			
								–0.79^60^			

a Measurement conditions: electrolyte, NBu_4_ClO_4_, *c* = 0.1 M; dry CH_2_Cl_2_; potential sweep rate, 100 mV s^–1^; working electrode, glassy carbon (GC); auxiliary electrode, Pt wire; reference electrode, Ag/AgCl. All measurements were conducted at room temperature.

b Ionization potential.

c Electron affinity.

The first oxidation of Cor-BiPh is irreversible in dichloromethane (DCM) and occurs at *E*_ox_^PA^ = 0.80 V, accompanied by two subsequent oxidations at *E*_ox_^PA^ = 0.96 V and *E*_ox_^PA^ = 1.04 V (Figure S6). The reduction for Cor-BiPh is reversible *E*_red_^PA^ – 0.70 V with *E*_red_^1/2^ = −0.83 V. Similar oxidation patterns were identified for Cor-Ph-PDI, featuring the first irreversible oxidation at *E*_ox_^PA^ = 0.87 V and subsequent oxidations at *E*_ox_^PA^ = 1.02 V and *E*_ox_^PA^ = 1.21 V. In the case of Cor-Ph-PDI, both reductions are associated with the PDI moiety, and their reversible reduction potential values are *E*_red_^PA^ = −0.56 V and *E*_red_^PA^ = −0.8 V, which corresponds to half-wave oxidation potentials at *E*_ox_^1/2^ = −0.67 V and *E*_ox_^1/2^ = −0.90 V, respectively.

Since both the first oxidation potential of corrole scaffold in Cor-Ph-PDI (+0.87 V) is slightly different than that for model Cor-BiPh (+0.80 V) and the first reduction potential of PDI moiety (−0.67 V) is different than that for PDI itself (−0.58 V), one can assume that the electronic coupling of the components of the dyad is very weak but still present.

A spectroscopic and photophysical investigation was carried out on two models, i.e., PDI, Cor-BiPh, as well as on the Cor-Ph-PDI dyad. The steady-state UV–vis absorption and emission spectra of PDI, Cor-BiPh, and the Cor-Ph-PDI dyad in toluene are shown in Figure 2 (fluorescence excitation spectra are shown in the Supporting Information (SI)). As shown in Figure 2a, the model for PDI has distinct vibronic progressions, where λ_0–0_ = 527 nm, λ_0–1_ = 490 nm, and λ_0–2_ = 460 nm; the model Cor-BiPh shows its Q-bands spreading between 500 and 650 nm and a Soret band stretching between 380 and around 440 nm. The Cor-Ph-PDI dyad displays spectra which are essentially the superimposed absorption spectra of the component models.

**Figure 2:**
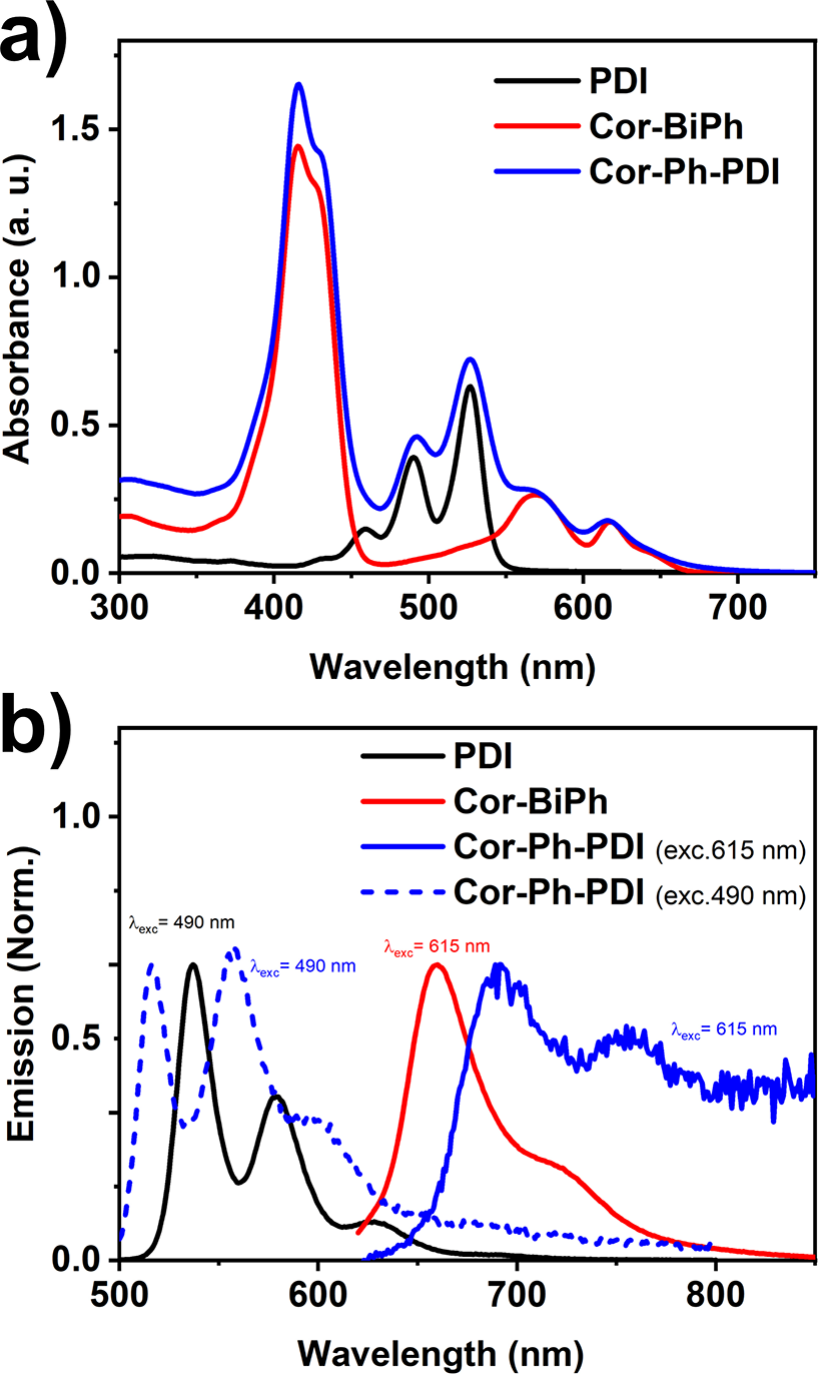
Steady-state UV–vis absorption (a) and emission spectra (b) of PDI, Cor-BiPh, and the Cor-Ph-PDI dyad (excited at two different wavelengths) in toluene.

No charge transfer bands were observed between the PDI and corrole units, and the additive properties of the spectra indicate very weak donor–acceptor electronic coupling. The emission spectra of models PDI and Cor-BiPh in toluene respectively excited at 490 and 615 nm and the Cor-Ph-PDI dyad excited at both 490 and 615 nm are shown in Figure 2b.

When the PDI model is excited at 490 nm, there are three emission bands (λ_0–0_ = 540 nm, λ_0–1_ = 580 nm, and λ_0–2_ = 630 nm), whereas when the Cor-BiPh chromophore is excited at 615 nm, there are two emission bands peaked at 657 and 720 nm. Upon excitation of the Cor-Ph-PDI dyad in toluene at 490 nm emission, the PDI unit prevails, which is however hypsochromically shifted by about 20 nm (Figure 2b). The emission originating from the corrole unit is much weaker. Interestingly, when the Cor-Ph-PDI dyad is excited at 615 nm, there is one weak emission band peaked at 682 nm, which is again bathochromically shifted versus that of corrole. The fluorescence quantum yields of PDI and Cor-BiPh are 0.83 and 0.08 respectively, whereas the fluorescence quantum yield of the Cor-Ph-PDI dyad is sharply decreased to ≈0.01, indicating the existence of ultrafast nonradiative decay pathways. Similar behavior was observed in CH_2_Cl_2_ and in benzonitrile (Table 2). Compared to C3-PI^61^ possessing corrole and PDI linked though an imide bond (hence electronically disconnected and separated by 25 Å), excitation of PDI in Cor-Ph-PDI leads to less pronounced corrole emission, whereas excitation of the corrole scaffold obviously leads to exclusive corrole-based emission, albeit bathochromically shifted. The emission lifetimes of reference PDI and Cor-BiPh in toluene at room temperature are 4.4 and 3.8 ns, respectively, whereas the emission lifetime of the Cor-Ph-PDI dyad cannot be monitored by TCSPC because of the very low fluorescence quantum yield.

**Table 2:** Photophysical Data for Dyes PDI, Cor-BiPh, and Cor-Ph-PDI

	Toluene			DCM			Benzonitrile		
Dye	λ_abs max_ (nm)	λ_em max_ (nm)	Φ_fl_	λ_abs max_ (nm)	λ_em max_ (nm)	Φ_fl_	λ_abs max_ (nm)	λ_em max_ (nm)	Φ_fl_
PDI	527	537	0.83^a^	524	533	0.88^a^	529	540	0.82^a^
Cor-BiPh	640	657,720	0.08^b^	639	656	0.06^b^	634	649	0.13^b^
Cor-Ph-PDI	415	516	0.010^a^	416	514	0.007^a^	432	520	0.006^a^
	492	558	0.0003^b^	493	556	0.00004^b^	497	562	0.0001^b^
	526	597		526	601		532	608	
	567	682		613	678		631		
	615	692			732				
		755							

a Rh6G in EtOH as a standard (excitation, 490 nm).

b Oxazine1 in EtOH as a standard (excitation, 615 nm).

The crossing points of the normalized steady-state absorption and emission spectra yield the lowest S_1_ energy of the Cor-Ph-PDI dyad, *E*_S_ = 1.82 eV. Our electrochemical data (Table 1) are as follows: for PDI moiety in Cor-Ph-PDI the first reduction potential is −0.67 V versus Ag/AgCl, while the first oxidation potential of corrole moiety is +0.87 V versus Ag/AgCl. Regarding its thermodynamic driving force, by using the Weller equation (eq 1) the change in Gibbs’s free energy can be calculated in different solvents.
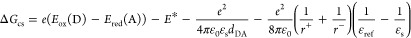
1where *E*_ox_ and *E*_red_ are the first oxidation and reduction potentials of donor and acceptor, respectively, E* is the energy approximated with the cross-point of absorption and fluorescence emission wavelength, the fourth term accounts for the Coulombic interactions between two ions produced at distance *d*_DA_ (4.83 Å) and screened by the solvent with a static dielectric constant *ε*_s_ (toluene, 2.4), *r*^*+*^ and *r*^*–*^ represent the effective ionic radii of donor (8.91 Å) and acceptor (9.07 Å) radical cation and anion, respectively, and *ε*_ref_ is the dielectric constant of the solvent used in electrochemistry (benzonitrile, 25.5). The calculated changes in free energy for the photoinduced electron transfer are −0.92 eV in toluene. This is a much larger value compared with those of both C3-PI^61^ (Δ*G*_CS_ = −0.05 eV) and Cor-(Ala)_4_-PDI^45^ (Δ*G*_ET_ = −0.2 eV) which places the process deep into the Marcus inverted region. The Weller analysis clearly suggests that the photoinduced electron transfer among the Cor-Ph-PDI dyad is energetically favored in toluene, exactly an exergonic process.

Electrochemical and photophysical analyses of PDI, Cor-BiPh, and the Cor-Ph-PDI dyad reveal favorable thermodynamics for the formation of charge separated (CS) state PDI^•–^-Cor^•+^. Consequently, we turned to femtosecond transient absorption (TA) spectroscopy to explore the excited-state dynamics for PDI, Cor-BiPh, and the Cor-Ph-PDI dyad. As shown in Figure 3a, photoexcitation of Cor-BiPh at 400 nm in toluene results in the population of its singlet in the first 10 ps, as indicated by ground-state bleach (GSB) at 572 nm, stimulated emission (SE) at 622 nm, and excited-state absorption (ESA) bands at 500 and 730 nm. With time increasing both ESA peaks have a blue shift, which are typical ESA of triplet excited-state transients of corroles, the lack of significant decrease in Δ*A* of the 450–550 nm TA band suggests comparable molar extinction coefficients of its singlet and triplet. Meanwhile the newly formed negative peak at 656 nm matches the steady-state fluorescence (Figure 2b); as shown in Figure 4a, the kinetics at around 650 nm shows a 11 ps increase, followed by a 3.8 ns decrease, two time constants are respectively related to the tautomerization^63^ and intersystem crossing (ISC) process. As shown in Figure 3b), PDI photoexcitation in toluene at 500 nm is followed by a GSB, SE, and a broad ESA signal from 650 to 800 nm peaked at 680 nm, attributable to a singlet excited state, with 4.5 ns lifetime (Figure 4b). For the Cor-Ph-PDI dyad, upon photoexcitation at 400 and 500 nm in toluene (Figure 3c,d), we observe that the fsTA features of PDI singlet appear and then rapidly convert to those of PDI^•–^ (ESA peaked at 720 nm), indicating an electron transfer process, and there is no obvious indication of the presence of Cor^•+^ even with excitation wavelength at 500 nm. The transient absorption spectra are marked by robust maxima at 710 nm, a characteristic ascribed to the perylenediimide radical anion based on extensive earlier studies.^64,65^ The rate constant of the electron transfer process from corrole to PDI and the corresponding charge recombination can be generated by fitting the rise and decay part of kinetic traces at 720 nm. Importantly, no signal originating from the corrole-oxidized radical can be detected. This observation aligns with expectations, as the corrole cation possesses a low molar absorption coefficient (10^4^), whereas PDI^•–^ exhibits a molar absorption coefficient of approximately 100000.^64,65^

**Figure 3:**
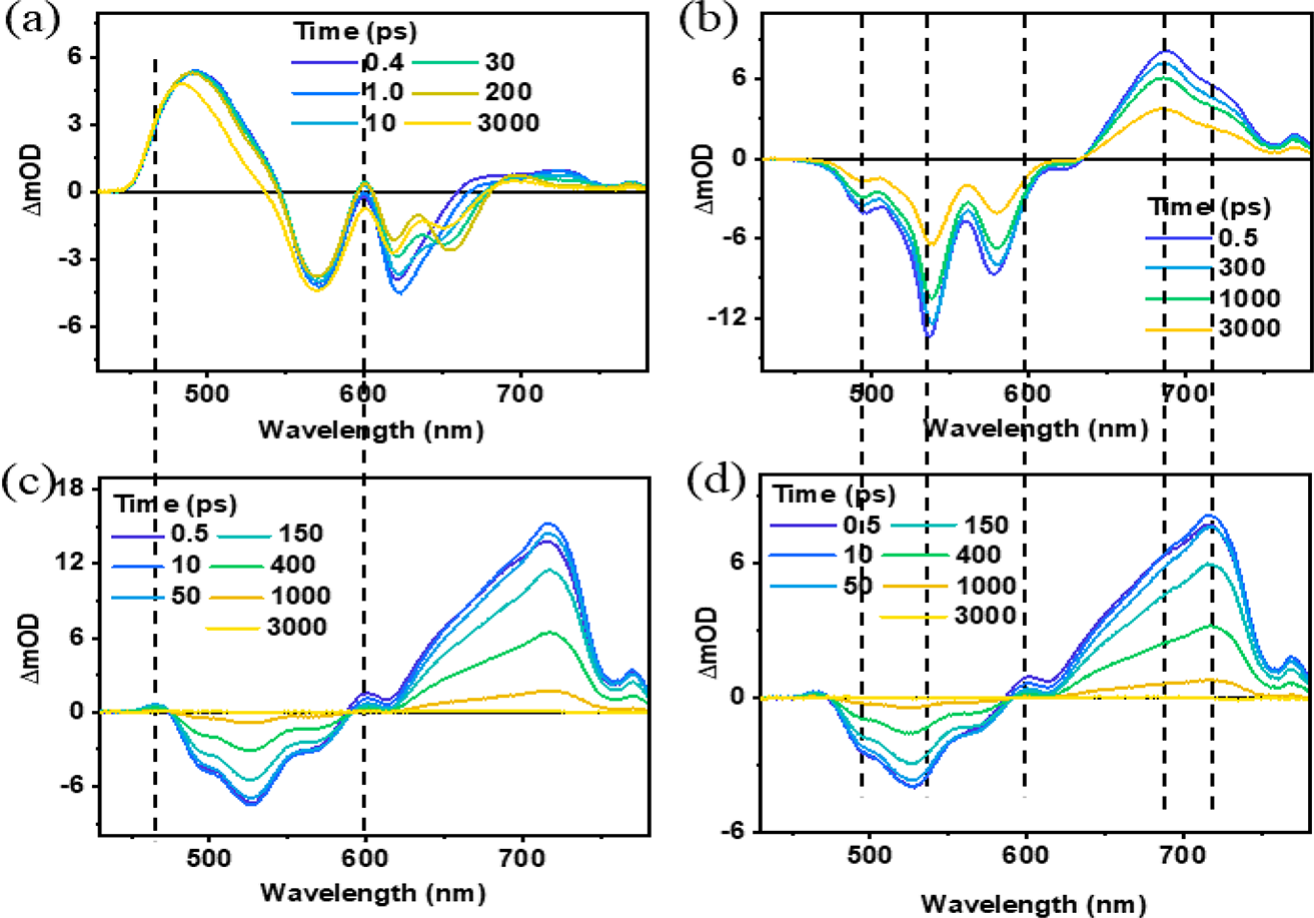
Transient absorption spectra for Cor-BiPh (a) and the Cor-Ph-PDI dyad (c) at λ_ex_ = 400 nm and for PDI (b) and the Cor-Ph-PDI dyad (d) at λ_ex_ = 500 nm in toluene.

**Figure 4:**
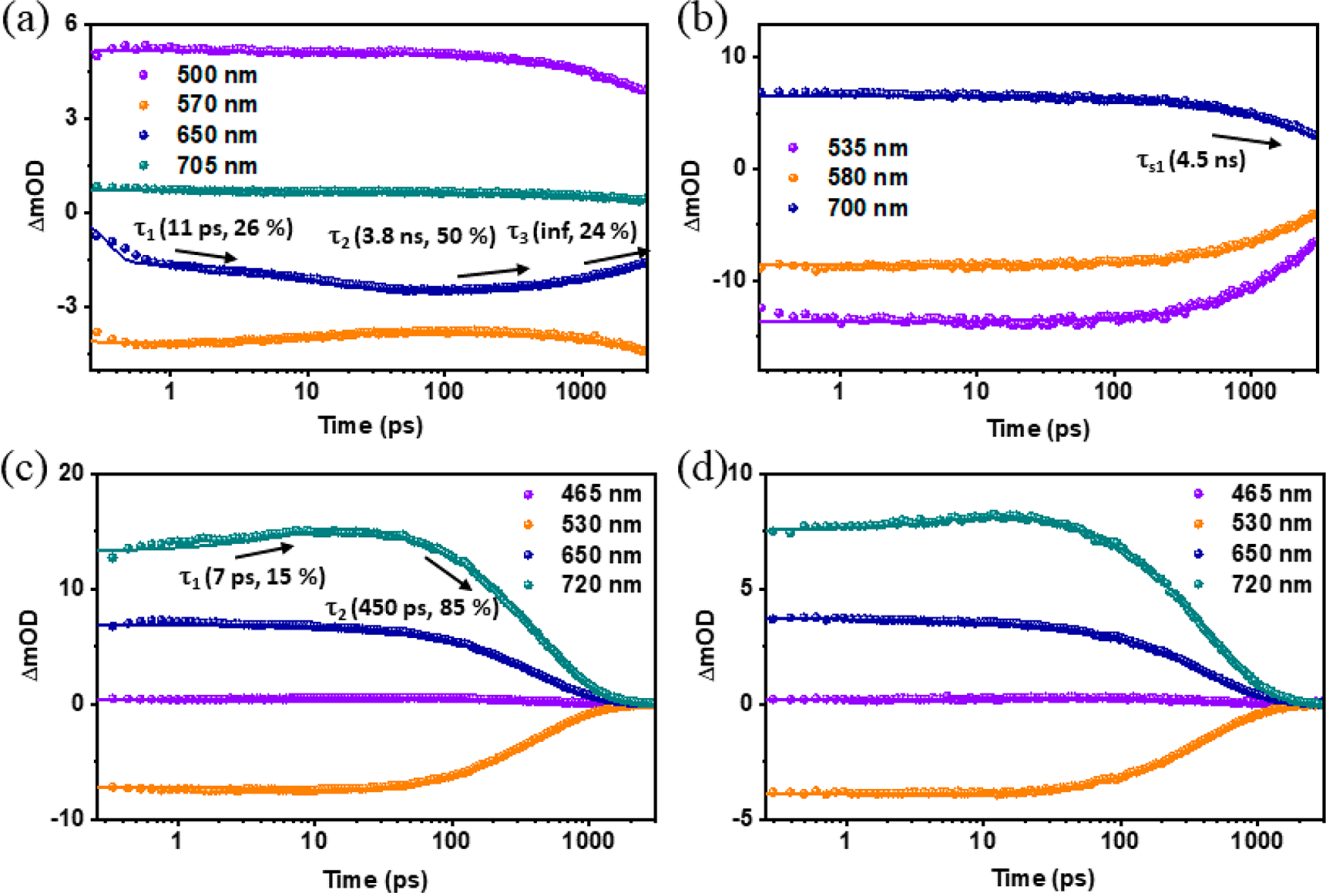
Transient absorption kinetics of Cor-BiPh (a) and the Cor-Ph-PDI dyad (c) at λ_ex_ = 400 nm and for PDI (b) and the Cor-Ph-PDI dyad (d) at λ_ex_ = 500 nm in toluene.

Thus, excitation of the corrole unit (400 nm) leads to hole transfer, whereas excitation of the PDI unit (500 nm) leads to an electron transfer to form a charge separated state as shown on the simplified Jablonski diagram (Figure 5). After the electron transfer hole is localized on the corrole moiety and electron on the PDI moiety.

**Figure 5:**
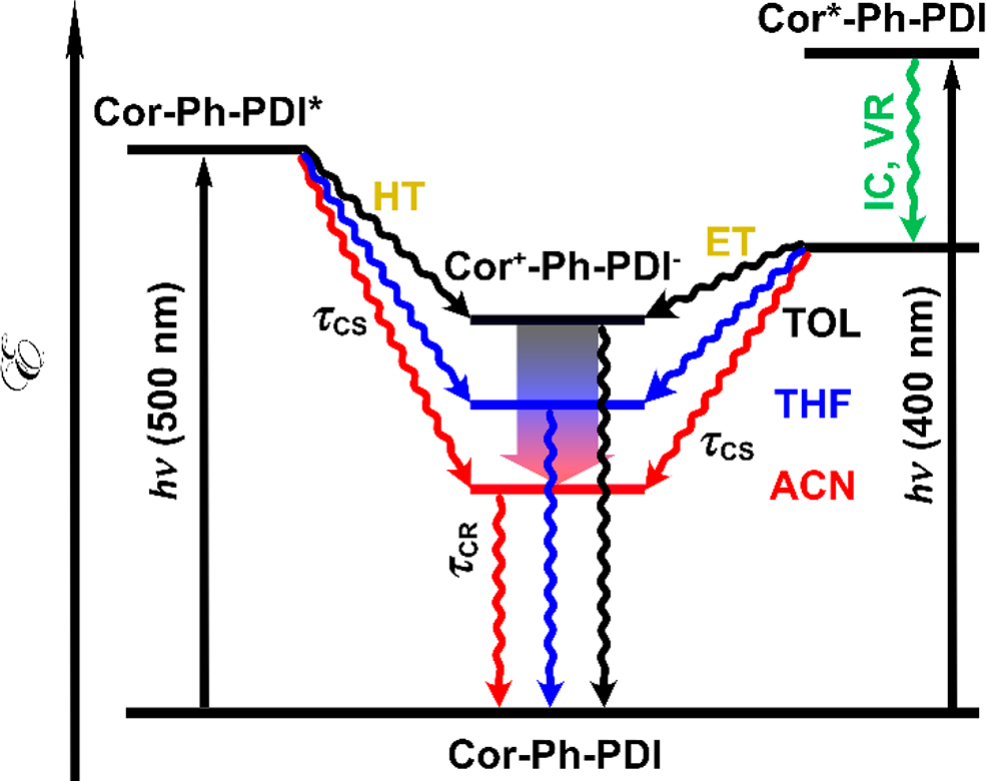
Jablonski diagram depicting photoinduced electron transfer and recombination process in the Cor-Ph-PDI dyad.

It is found that the electron transfer and the corresponding recombination process become faster in more polar solvents (Figure 4c,d and Figures S4 and S5): time constants for the electron transfer process from toluene, THF, to ACN are 7, 0.7, and 0.2 ps, respectively; time constants for the charge recombination process from toluene, THF, to ACN are 450, 12, and 2.4 ps, respectively. Consequently, *k*_ET_ is 30 times faster and the lifetime of the CS state is 5 times smaller than in the case of C3-PI.^61^

Corrole macrocycle and PDI scaffolds in the molecular structure of the dyad Cor-Ph-PDI obtained at the MP2/def2-SVP level of theory are roughly in perpendicular orientation to each other, with a center-to-center distance of about 7.7 Å and an angle between the mean planes of each chromophore of 74° (Figure 6a). An electron-rich corrole core retains essentially the same significantly distorted geometry, originating from the close proximity of the inner hydrogens, as in many previously characterized free-base corroles.^56−58^ In contrast, the PDI scaffold is slightly twisted (Figure 6a). Expectedly, peripheral −C_6_F_5_ groups are predicted to be out of the plane of the corrole moiety. Interestingly however, in the absence of such, all peripheral groups (i.e., C_6_F_5_ and C_13_H_27_), the corrole macrocycle and PDI moiety would stack, possibly dramatically increasing the through-space electron communication between the dyad’s donor and acceptor units, as can be seen in Figure S1.

**Figure 6:**
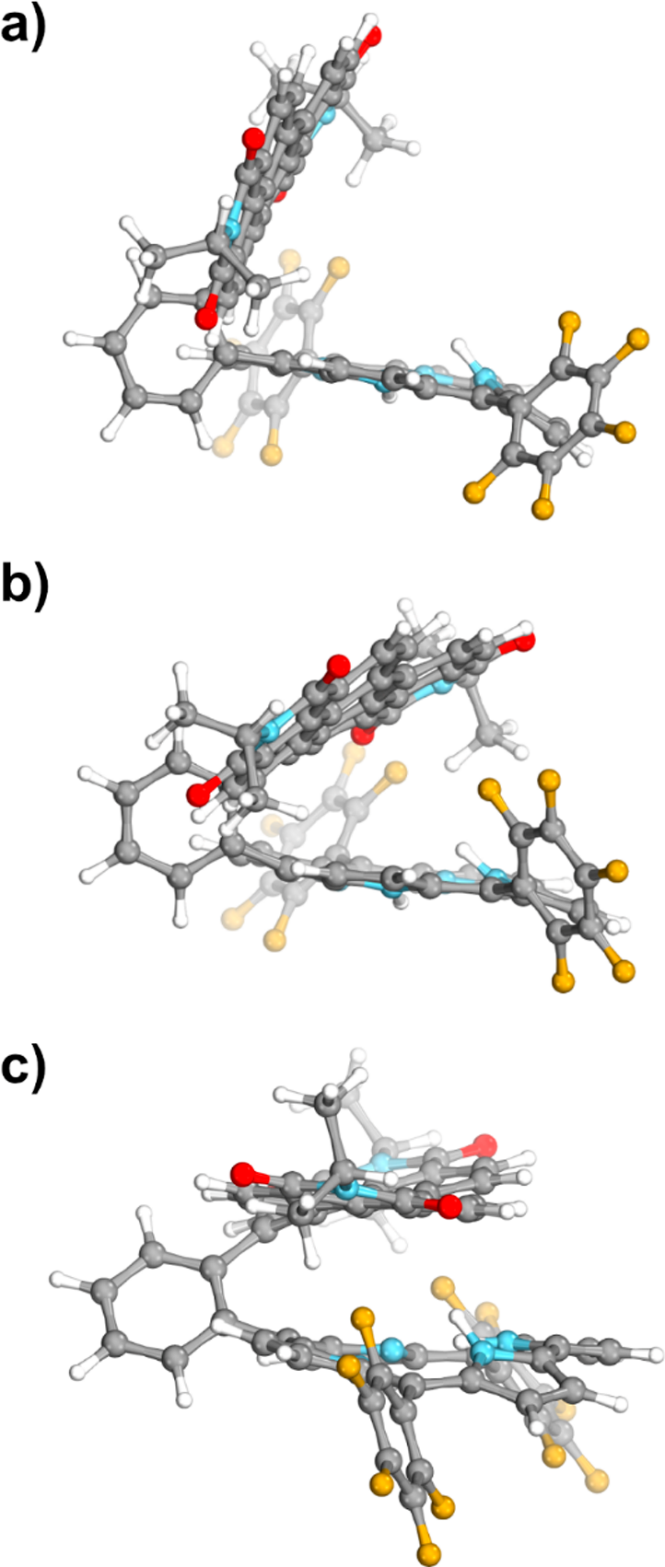
Molecular structures for the ground state (S_0_) obtained at (a) MP2/def2-SVP and (b) PW6B95-D3/6-31G(d,p). (c) The latter method was also used to optimize the first singlet excited state (S_1_).

The lowest five vertical excited states were computed at ADC(2)/def2-SVP//MP2/def2-SVP for Cor-Ph-PDI and Cor-Ph and PDI components separately (Table S1). In line with the experimental observations, low-lying electronic excitations of the dyad are composed of two sets of bright locally excited states; transitions at lower energy can be assigned to the excitation of the corrole scaffold, while at higher energy, transitions are expected to be due to excitation of perylenediimide. This effect is illustrated in Figure S2, where we superimpose excitation energies computed for the dyad and its separated units. Our calculations also predict the presence of two CS states of the dyad with excitation energies around 2.7 and 3.0 eV (their dipole moments are higher than 25 D and fragment charge differences are approximately 0.9). For these states, an electron transfer from the corrole to the PDI would occur, and these transitions have significantly lower oscillator strength than the bright LE states previously mentioned. For each transition, we computed the respective molecular orbitals (Figure 7) and the natural transition orbitals (Figure S2). Both results suggest that the electron density is essentially localized on the corrole and PDI units.

**Figure 7:**
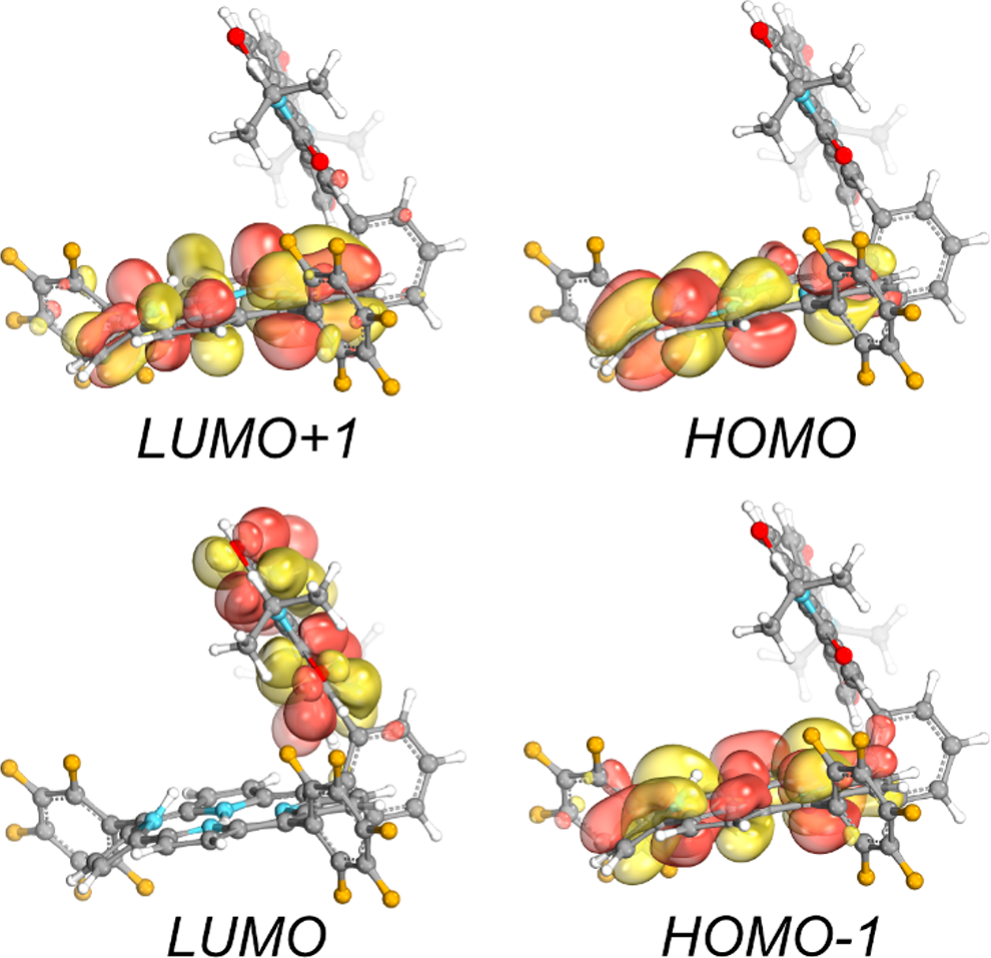
Molecular orbitals involved in bright electronic excitations of the dyad.

Ground-state geometry was obtained using two levels of theory: ab initio (MP2/def2-SVP) and DFT using the density functional PW6B95D3. With respect to the ab initio geometry, DFT seems to significantly favour the stacking between both units. The angle between PDI and corrole units decreases by about 30° with PW6B95D3/6-31G(d,p). Vertical excitation energies were also computed using the same density functional and additionally using the ADC2(2)/def2-SVP level of theory. The obtained results indicate that TDDFT overstabilizes the CT states by about 1.6 eV. Excited-state geometry optimization at the ADC(2) level of theory was not feasible due to the size of the dyad molecule. However, comparing both ab initio (MP2) and TD-DFT/PW6B95D3 geometries in the ground state (Figure 6), we can expect a larger through-space electronic coupling between the corrole and PDI units. With respect to the first singlet (S_1_) excited-state optimization was performed at the PW6B95D3/6-31G(d,p) level of theory (Table S1b), the molecule presents both PDI and corrole units stacked despite the peripheral bulky groups, separated by about 4.8 Å (Figure 6c).

Such proximity between these units allows an electron transfer from the corrole to the PDI moieties, resulting in the population of a non-emissive charge-separated (CS) state with fluorescence energy estimated to be about 1 eV. However, the CS state may be significantly overstabilized due to the artifacts of DFT in accurately describing electronic transitions involving molecular orbitals separated by large spatial extents.

In conclusion, we proved that meso-substituted corrole can be efficiently prepared, even from exceptionally sterically encumbered aldehyde. Both electrochemical and spectroscopic studies confirmed a weak but not-zero coupling of the components in the resulting dyad in the ground state, which is characterized by an intense and extended collection of light throughout the whole visible region. Photophysical studies supported by ab initio and DFT-based computations and femtosecond transient absorption measurements revealed that the efficient charge-separated state is induced by excited-state dihedral motion. The dihedral reduction between the two moieties, which breaks the large angle and long distance arrangement in the ground state and increases the level of interchromophoric electronic coupling, plays a decisive role during the electron/hole transfer process. This work showcases the importance of interchromophoric torsion motion in the excited state of a weakly coupled dyad system to enable efficient electron transfer.

## Supporting Information Available

Details of the synthetic procedures; characterization details; ^1^H, ^13^C{^1^H} NMR, and HRMS spectra; computational studies; photophysical measurements; electrochemistry; additional figures as mentioned in the text (PDF)
Cartesian coordinates (PDF)
Transparent Peer Review report available (PDF)
