# Supplementary material for: Efficient Electron Transfer Driven by Excited-State Structural Relaxation in Corrole–Perylenedimiide Dyad
Source: J Phys Chem Lett. 2024 May 8;15(19):5231–8. doi: 10.1021/acs.jpclett.4c00916 (PMC11103693; doi:10.1021/acs.jpclett.4c00916)
Supplement: Supplementary file 1 — jz4c00916_si_001.pdf [file jz4c00916_si_001.pdf]

Supporting Information

for

Efficient Electron Transfer Driven by Excited State Structural Relaxation in Corrole-Perylenedimiide Dyad

Damian Kusy,[1] Hongwei Song,[2] Antoni Rząca,[1,3] Marzena Banasiewicz,[4] Cristina A. Barboza,[4,5]* Dongho Kim,[2]* Daniel T. Gryko[1]*

[1] Institute of Organic Chemistry, Polish Academy of Sciences, Kasprzaka 44/52 01-224 Warsaw, Poland.

[2] Spectroscopy Laboratory for Functional π-Electronic Systems and Department of Chemistry, Yonsei University, Seoul 03722, Republic of Korea

[3] Warsaw University, Faculty of Chemistry, Pasteura 1, 02-093 Warsaw, Poland.

[4] Institute of the Physics of Polish Academy of Sciences, Al. Lotników 32/46, 02-668 Warsaw, Poland.

[5] Institute of Advanced Materials, Faculty of Chemistry, Wrocław University of Science and Technology, Wybrzeże Wyspiańskiego 27, Wrocław, 50-370, Poland

E-mail : dtgryko@icho.edu.pl
E-mail: dongho@yonsei.ac.kr
E-mail: crissetubal@ifpan.edu.pl

Contents

1. General remarks 3
2. Experimental section 4
2.1. Synthetic procedure for the synthesis of PDI-Ph-CHO 4
2.2. Synthetic procedure for the synthesis of BiPh-CHO 5
2.3. Synthetic procedure for the synthesis of Cor-Ph-PDI 5
2.4. Synthetic procedure for the synthesis of Cor-BiPh 6
3. Computational studies 7
4. Photophysical measurements 11
5. Electrochemistry 154
6. ^{1}H and ^{13} C NMR, LRMS, HRMS spectral data 176
7. References 209

1. General remarks

All reagents and solvents were purchased from commercial sources and were used as received unless otherwise noted. For water-sensitive reactions solvents were dried using Solvent Purification System from MBraun (https://www.mbraun.com/us/). Reactions involving moisture and oxygen sensitive compounds were performed under a stream of argon. The reaction progress was monitored by means of thin layer chromatography (TLC), which was performed on aluminium foil plates, covered with Silica gel 60 F254 (Merck). Products purification was done by means of column chromatography with Kieselgel 60 (200-400 mesh, Merck). The identity and purity of prepared compounds were proved by ^{1}H NMR and ^{13}C NMR spectrometry as well as by MS-spectrometry (via EI-MS , ESI-MS or APCI-MS). NMR spectra were measured on Bruker AM 500 MHz, Varian 600 MHz, Varian 500 MHz instruments with TMS as internal standard. Chemical shifts for ^{1}H NMR are expressed in parts per million (ppm) relative to tetramethylsilane (δ 0.00 ppm), $CDCl_3$ (δ 7.26 ppm) CD_2Cl_2 (δ 5.33 ppm). Chemical shifts for ^{13}C NMR are expressed in ppm relative to $CDCl_3$ (δ 77.16 ppm), CD_2Cl_2 (53.84 ppm). Data are reported as follows: chemical shift, multiplicity (s = singlet, bs = broad singlet, d = doublet, dd = doublet of doublets, ddd = doublet of doublet of doublets, t = triplet, td = triplet of doublets, q = quartet, qu = quintet, m = multiplet), coupling constant (Hz), and integration.

2. Experimental section

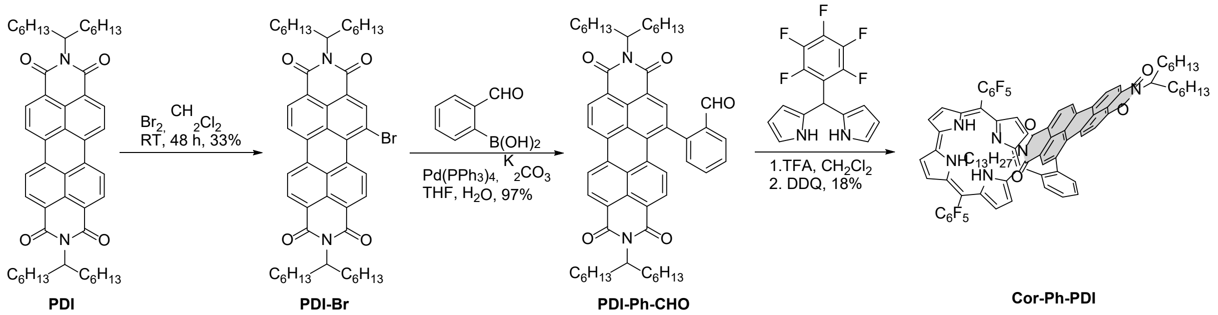

Scheme S1. Synthesis of **PDI-Ph-CHO** and **Cor-Ph-PDI**.

N,N'-Di-(1-hexylheptyl)-perylene-3,4,9,10-tetracarboxydianhydride: (**PDI)** was prepared according to the literature procedure [1]

N,N'-Di-(1-hexylheptyl)-1-bromoperylene-3,4,9,10-tetracarboxydianhydride: (**PDI-Br)** was prepared according to the literature procedure[2,3]

2.1. Synthetic procedure for the synthesis of PDI-Ph-CHO

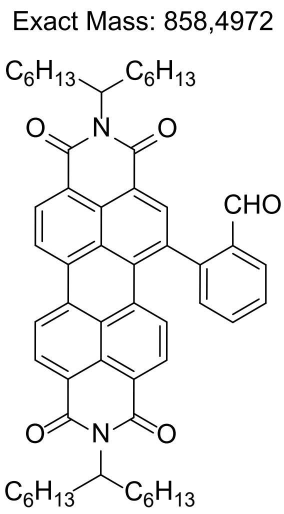

PDI-Ph-CHO

A Schlenk flask was equipped with a magnetic stir bar and connected to an argon/vacuum line. The reaction setup was backfilled with argon. PDI-Br (0.4g, 0.48 mmol, 1 eq), $Pd(PPh_3)_4$ (27.7 mg, 0.024 mmol, 0.05 eq), and (2-formylphenyl)boronic acid (108 mg, 0.719 mmol, 1.5 eq) were added to the flask and dissolved in 10 mL of THF. K_2CO_3 (200 mg, 1.44 mmol, 3 eq) was dissolved in 2.5 mL of H_2O and then added to the reaction mixture. The vessel was carefully evacuated (until the mixture started to boil) and backfilled with argon (three times). The reaction was conducted at 60 °C for 12 hours. The reaction mixture was transferred to a separatory funnel, the organic layer was separated, and the water layer was extracted with dichloromethane (3 × 10 mL). The organic layers were combined. Solvents were evaporated, and the crude product was purified by column chromatography (hexane/ethyl acetate 9:1).

2-(1,3,8,10-tetraoxo-2,9-di(tridecan-7-yl)-1,2,3,8,9,10-hexahydroanthra[2,1,9-def:6,5,10-d'e'f']diisoquinolin-5-yl)benzaldehyde: PDI-PhCHO was obtained as red solid yield 400 mg, 4.66 mmol, (97%). **^{1}H NMR (500 MHz, $CDCl_3$)** δ 9.91 (s, 1H), 8.78 – 8.59 (m, 4H), 8.50 –

8.44 (m, 1H), 8.16 – 8.11 (m, 2H), 7.84 (t, $^3J_{HH}$ = 7.5 Hz, 1H), 7.74 (t, $^3J_{HH}$ = 7.5 Hz, 1H), 7.57 (d, $^3J_{HH}$ = 8.3 Hz, 1H), 7.52 (d, $^3J_{HH}$ = 7.5 Hz, 1H), 5.23 – 5.04 (m, 2H), 2.37 – 2.08 (m, 4H), 1.91 – 1.70 (m, 4H), 1.42 – 1.09 (m, 32H), 0.90 – 0.71 (m, 12H). **^{13}C NMR (126 MHz, $CDCl_3$)** δ: 191.01, 145.10, 137.67, 136.05, 134.39, 133.61, 132.73, 131.73, 130.66, 129.58, 129.39, 129.33, 129.07, 127.47, 123.85, 123.15, 55.02, 54.86, 32.50, 32.47, 31.90, 31.87, 29.36, 29.34, 27.05, 27.01, 22.73, 22.72, 14.18, 14.17.

2.2. Synthetic procedure for the synthesis of BiPh-CHO

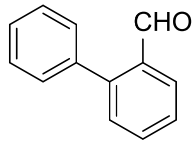

BiPh-CHO

A Schlenk flask, equipped with a magnetic stir bar and connected to an argon/vacuum line, was backfilled with argon. Bromophenol (0.84 mL, 8.0 mmol, 1 eq), (2-formylphenyl)boronic acid (1.8 g, 12.0 mmol, 1.5 eq), and $Pd(PPh_3)_4$ (462 mg, 0.40 mmol, 0.05 eq) were added to the flask and dissolved in 160 mL of THF. K_2CO_3 (3.32 g, 24.0 mmol, 3 eq) was dissolved in 40 mL of H_2O and then added to the reaction mixture. The vessel was carefully evacuated (until the mixture started to boil) and backfilled with argon (three times). The reaction was conducted at 60°C for 12 hours. After completion, the reaction mixture was transferred to a separatory funnel, the organic layer separated, and the water layer extracted with dichloromethane (3 x 160 mL). The organic layers were combined. Solvents were evaporated, and the crude product was purified by column chromatography (hexane/ethyl acetate 9:1). The structure confirmed by NMR and mass spectrometry. The spectrum is consistent with the literature. The product is also commercially available.

2.3. Synthetic procedure for the synthesis of Cor-Ph-PDI

Exact Mass: 1458,5768

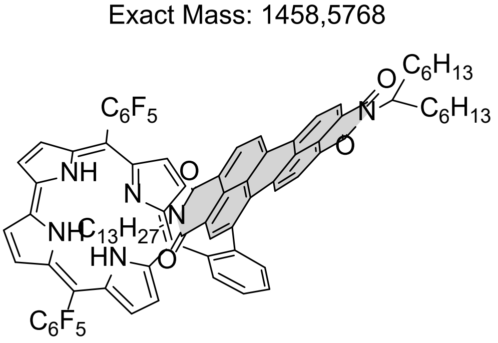

Cor-Ph-PDI

PDI-PhCHO (700 mg, 0.81 mmol, 1.0 equiv.) and 508 mg (1.62 mmol, 2.0 equiv.) of pentafluorophenyldipyrromethane were dissolved in 60 ml of DCM. To this solution, 37 µl (0.32 mmol, 0.4 equiv.) of TFA were added, and the mixture was stirred for 24 hours at room temperature. Subsequently, the reaction mixture was diluted to 500 ml with DCM, and 555 mg of DDQ (2.44 mmol, 3 equiv.) dissolved in 5 ml of toluene were added. The solution was stirred for an additional 3 hours. To isolate the product, the reaction mixture was initially passed through silica gel, and the filtrate was evaporated to dryness with Celite. Column chromatography (0 → 100%

DCM/hexanes) and crystallization from MeOH were performed, resulting in the yield of 212 mg of **Cor-Ph-PDI**, 0.145 mmol, (17.8%).

5-((S)-2-((9s,19s)-9,19-bis(perfluorophenyl)-20H-porphyrin-14-yl)phenyl)-2,9-di(tridecan-7-yl)anthra[2,1,9-def:6,5,10-d'e'f']diisoquinoline-1,3,8,10(2H,9H)-tetraone: **Cor-Ph-PDI** was obtained as black solid yield 212 mg (17.8%). **^{1}H NMR (600 MHz, Methylene Chloride-*d*$_2$)** δ 8.99 (d, $^3J_{HH}$ = 4.2 Hz, 1H), 8.94 (d, $^3J_{HH}$ = 3.6 Hz, 1H), 8.75 – 8.62 (m, 2H), 8.49 – 8.25 (m, 7H), 8.19 (t, $^3J_{HH}$ = 7.8 Hz, 1H), 8.06 – 7.98 (m, 2H), 7.93 – 7.87 (m, 1H), 7.82 – 7.72 (m, 2H), 7.37 – 7.29 (m, 1H), 7.29 – 7.18 (m, 1H), 5.25 – 5.07 (m, 1H), 4.96 – 4.80 (m, 1H), 2.33 – 1.61 (m, 5H), 1.60 – 1.05 (m, 28H), 1.05 – 0.67 (m, 12H). **HRMS (APCI- negative)** calcd for $C_{87}H_{75}N_6O_4F_{10}$ 1457.5690 [M-H]$^-$, found: 1457.5685

2.4. Synthetic procedure for the synthesis of Cor-BiPh

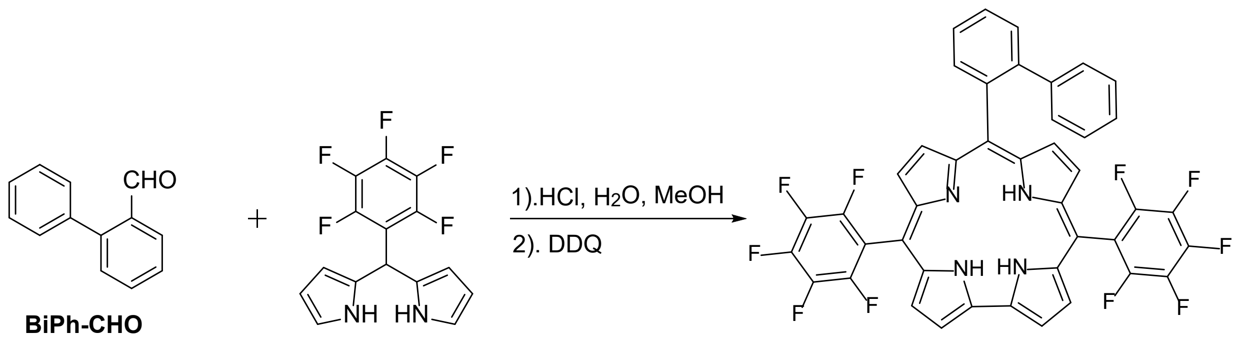

Scheme S2. Synthesis of **Cor-BiPh**.

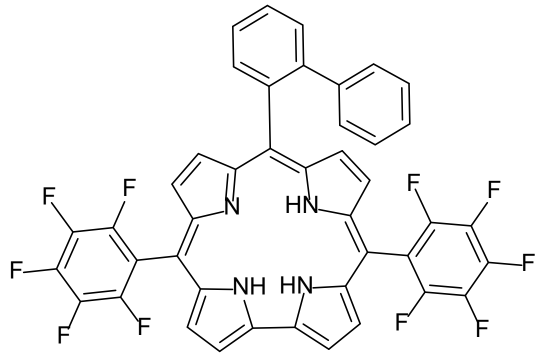

Biphenyl-2-carboxyaldehyde (**BiPh-CHO**) (182 mg, 1.0 mmol, 1 eq) and **pentafluorophenyldipyrromethane** (625 mg, 2.0 mmol, 2 eq) were dissolved in MeOH (100 mL). Subsequently, an HCl solution (36%, 5 mL) in H_2O (100 mL) was added, and the reaction stirred for 3 hours at room temperature. The mixture was then extracted with dichloromethane (200 mL), the organic layer dried over anhydrous Na_2SO_4, filtered, and diluted to 1.5 L with dichloromethane. A solution of DDQ (681 mg, 3.0 mmol, 3 eq) in toluene (20 mL) was added, and the reaction stirred for an additional 3 hours at room temperature. The resulting mixture was passed through silica gel, and the filtrate evaporated to dryness with Celite. Column chromatography (0 → 80% DCM/hexanes) and crystallization from MeOH afforded 111 mg of **Cor-BiPh** (0.14 mmol, 14% yield). **5-([1,1'-biphenyl]-2-yl)-10,19-bis(perfluorophenyl)-**

20H-porphyrin: **Cor-BiPh** was obtained as black solid yield 111 mg, (0.14 mmol), (14%). **^{1}H NMR (600 MHz, Methylene Chloride-*d*$_2$)** δ 9.11 (d, $^3J_{HH}$ = 4.2 Hz, 2H), 8.65 (s, 4H), 8.55 (s, 2H), 8.10 (dd, $^3J_{HH}$ = 7.5, 1.0 Hz, 1H), 7.88 (td, $^3J_{HH}$ = 7.6, $^4J_{HH}$ = 1.4 Hz, 1H), 7.82 (dd, $^3J_{HH}$ = 7.9, $^4J_{HH}$ = 1.2 Hz, 1H), 7.72 (td, $^3J_{HH}$ = 7.6, $^4J_{HH}$ = 1.4 Hz, 1H), 7.06 – 7.02 (m, 2H), 6.55 – 6.46 (m, 3H). **LRMS (APCI)** calcd for $C_{43}H_{20}F_{10}N_4$ 782.15 $[M+H]^+$, found: 783.16

3. Computational studies

Theoretical exploration was done using a model molecule, replacing the alkyl chains attached to the perylene with isopropyl groups attached to each nitrogen atom. The dyad and its fragments, corrole and PDI, were optimized at the second-order Møller-Plesset perturbation theory MP2/def2-SVP level of theory. The vertical excited states were computed using the second-order algebraic diagrammatic construction theory (ADC(2)) available in the software Turbomole version 7.7.0. Charge transfer was estimated using the transition density matrix analysis available in the software Theodore (reference: F. Plasser, "TheoDORE: A toolbox for a detailed and automated analysis of electronic excited state computations", J. Chem. Phys., (2020), 152, 084108) and molecular structures and orbitals were obtained using IboView (G. Knizia, Intrinsic atomic orbitals: An unbiased bridge between quantum theory and chemical concepts J. Chem. Theory Comput., 9 4834 (2013))

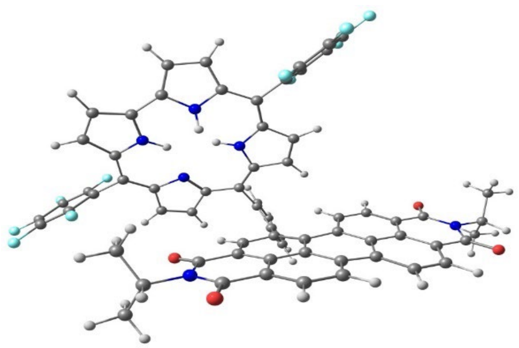

Dyad Cor-Ph-PDI

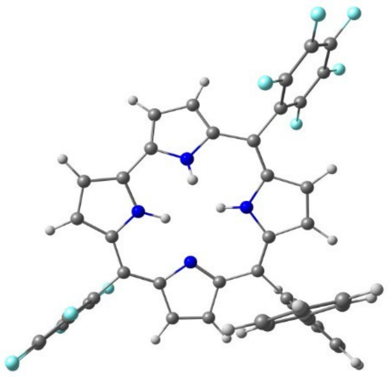

Corrole

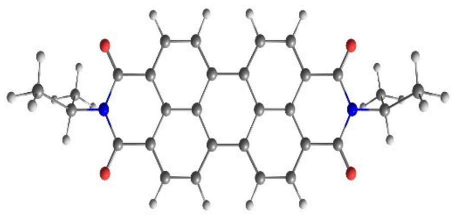

PDI

Figure S1. Molecular structure of the dyad, corrole, and PDI optimized at the MP2/def2-SVP level of theory.

Table S1a. Vertical transition energy (ΔE), oscillator strength (*f*), dipole moment (μ), and leading electronic configurations of dyad, corrole, and PDI computed with the ADC2/def2-SVP method at the MP2/def2-SVP equilibrium geometry of the ground state.

	State	E/eV	f	μ/D	Assignment	
		**PDI + Corrole**				
S_0		-	-	2.64	$(301)^2$	
S_1	$\pi\pi^*$	2.33	0.24	5.49	0.84(301-303)	LE (corrole)
S_2	$\pi\pi^*$	2.53	0.21	1.46	0.80(300-303)	LE (corrole)
S_3	$\pi\pi^*$	2.66	0.06	25.74	0.89(300-302)	CT (corrole-perylene)
S_4	$\pi\pi^*$	2.89	0.56	4.55	0.89(299-302)	LE (perylene)
S_5	$\pi\pi^*$	2.93	0.13	28.77	0.83(301-302)	CT (corrole-perylene)
		**Corrole**				
S_0		-	-	3.40	$(198)^2$	
S_1	$\pi\pi^*$	2.34	0.25	7.22	0.77(198-199)	LE
S_2	$\pi\pi^*$	2.52	0.26	1.25	0.76(197-199)	LE
S_3	$\pi\pi^*$	3.44	0.45	3.44	0.76(198-200)	LE
S_4	$\pi\pi^*$	3.61	0.81	3.65	0.75(197-200)	LE
S_5	$\pi\pi^*$	3.90	0.01	1.68	0.80(193-199)	LE
		**PDI**				
S_0		-	-	0.13	$(124)^2$	
S_1	$\pi\pi^*$	2.96	0.86	0.18	0.99(124-125)	LE
S_2	$\pi\pi^*$	3.78	0.00	0.15	0.80(123-125)	LE
S_3	$\pi\pi^*$	4.02	0.03	0.16	0.82(122-125)	LE
S_4	$\pi\pi^*$	4.18	0.00	0.19	0.79(120-125)	LE
S_5	$\pi\pi^*$	4.26	0.00	0.16	0.82(124-127)	LE

Table S1b. Natural transition orbitals corresponding to the lowest electronic excitations of the dyad, PDI, and corrole obtained at ADC(2)/def2-SVP

State	*Occupied*	*Virtual*	*State*	*Occupied*	*Virtual*
	**Dyad**			**Corrole**	

S_1 (LE)	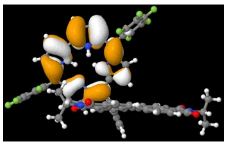	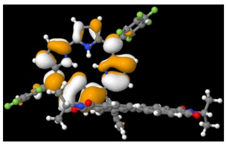	S_1 (LE)	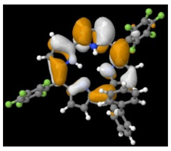	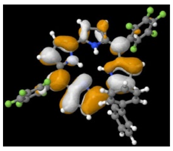
S_2(LE)	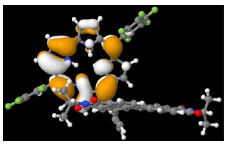	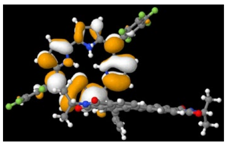	S_2 (LE)	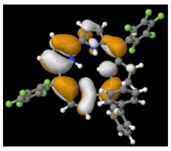	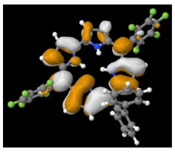
S_3(CT)	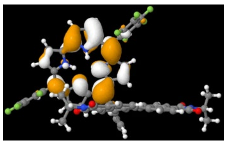	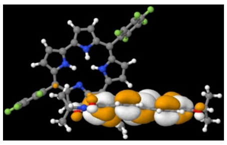	S_3 (LE)	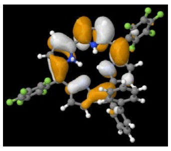	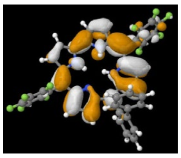
S_4(LE)	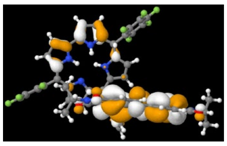	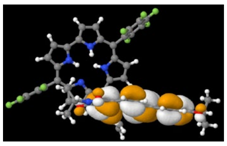	S_4 (LE)	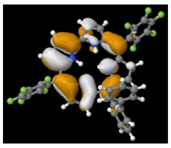	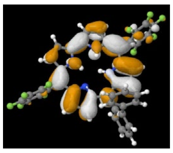
S_5(CT)	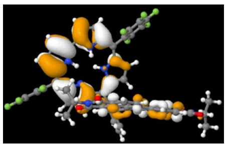	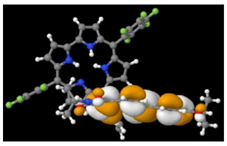	S_5 (LE)	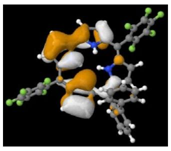	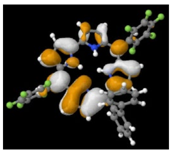
	**PDI**				
S_1 (LE)	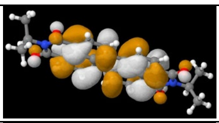	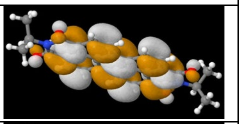			
S_2 (LE)	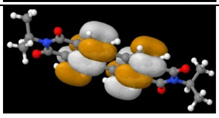	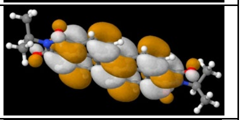			
S_3 (LE)	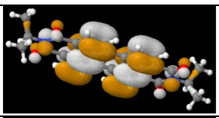	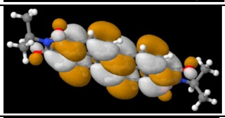			
S_4 (LE)	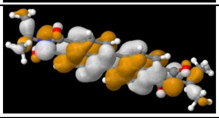	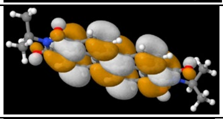			
S_5 (LE)	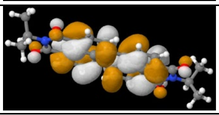	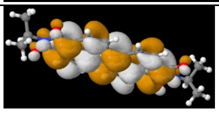			

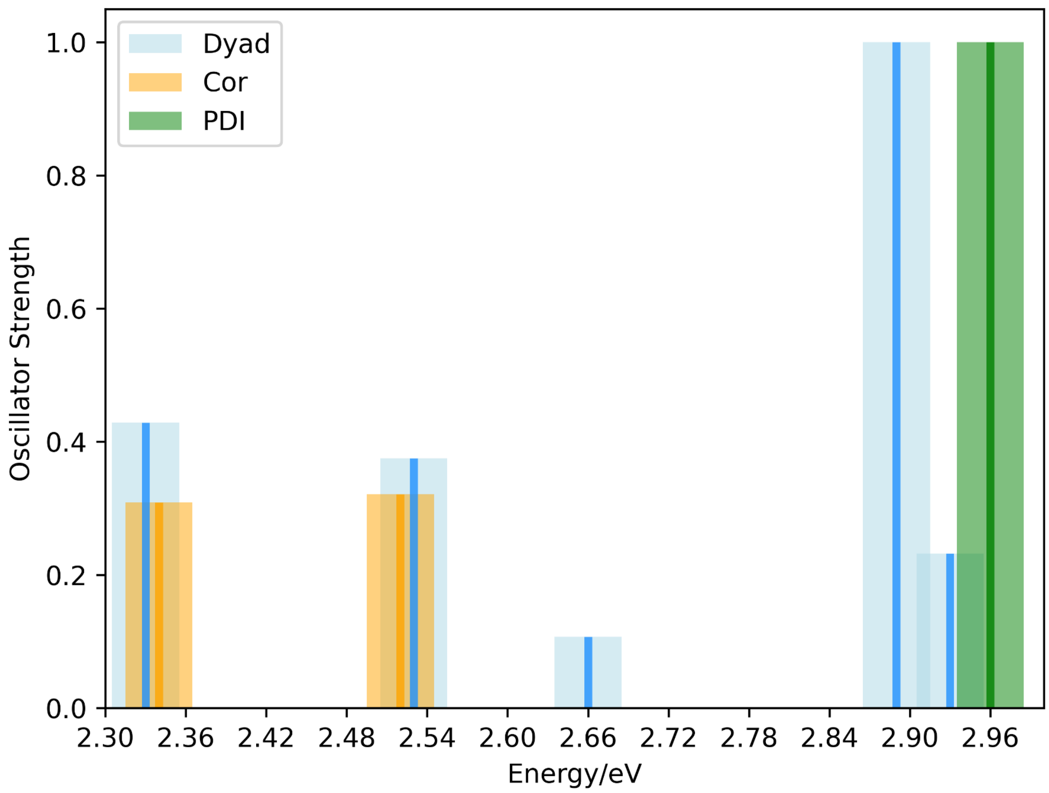

Figure S2. Comparison of lowest five vertical excited states obtained at ADC2/def2-SVP//MP2/def2-SVP level of theory for the dyad, corrole and PDI.

4. Photophysical measurements

Steady-State Spectroscopy: Steady-state absorption spectra were measured on a UV/Vis spectrometer (Varian, Cary5000) and photoluminescence spectra were measured on a fluorescence spectrophotometer (Edinburgh Instuments FLS1000). Anhydrous-grade solvents were purchased from Sigma-Aldrich and used without further purification. All steady-state measurements were carried out by using a quartz cuvette with a pathlength of 1 cm at ambient temperatures.

Femtosecond Transient Absorption Measurements: The femtosecond transient absorption (fs-TA) spectrometer consists of an optical parametric amplifier (OPA; Palitra, Quantronix) pumped by a Ti:sapphire regenerative amplifier system (Integra-C, Quantronix) operating at 1 kHz repetition rate and an optical detection system. The generated OPA pulses have a pulse width of ~ 200 fs in the range of 280-2700 nm, which are used as pump pulses. White light continuum (WLC) probe pulses were generated using a sapphire window (4 mm thick) by focusing a small portion of the fundamental 800 nm pulses which was picked off by a quartz plate before entering the OPA. The time delay between pump and probe beams was carefully

controlled by making the pump beam travel along a variable optical delay (ILS250, Newport). Intensities of the spectrally dispersed WLC probe pulses are monitored by a High-Speed Spectrometer (Ultrafast Systems) for both visible and near-infrared measurements. To obtain the time-resolved transient absorption difference signal (ΔA) at a specific time, the pump pulses were chopped at 500 Hz and absorption spectra intensities were saved alternately with or without pump pulse. Typically, 4000 pulses excite the samples to obtain the fs-TA spectra at each delay time. The polarization angle between pump and probe beam was set at the magic angle (54.7°) using a Glan-laser polarizer with 4 a half-wave retarder to prevent polarization-dependent signals. Cross-correlation fwhm in pump-probe experiments was around 200 fs and chirp of WLC probe pulses was measured to be 1.2 ps in the 450-800 nm region. To minimize chirp, all reflection optics were used in the probe beam path. A quartz cell of 2 mm path length was employed. After completing each set of TA experiments, the absorption spectra of all samples were carefully checked to rule out the presence of artifacts or spurious signals arising from, for example, degradation or photo-oxidation of the samples in question.

Time-correlated single photon counting measurements: A time-correlated single-photon-counting (TCSPC) system was used for measurements of spontaneous fluorescence decay. As an excitation light source, we used a mode-locked Ti:sapphire oscillator (Spectra Physics, MaiTai BB) which provides ultrashort pulse (center wavelength of 800 nm with 80 fs at FWHM) with high repetition rate (80 MHz). This high repetition rate was reduced to 800 kHz by using homemade pulse-picker. The pulse-picked output was frequency doubled by a 1-mm-thick BBO crystal (type-I, θ = 29.2°, EKSMA). The fluorescence was collected by a microchannel plate photomultiplier (MCP-PMT, Hamamatsu, R3809U-51) with a thermoelectric cooler (Hamamatsu, C4878) connected to a TCSPC board (Becker & Hickel SPC-130). The overall instrumental response function was about 25 ps (FWHM). A vertically polarized pump pulse by a Glanlaser polarizer was irradiated to samples, and a sheet polarizer set at an angle complementary to the magic angle (54.7°), was placed in the fluorescence collection path to obtain polarization-independent fluorescence decays.

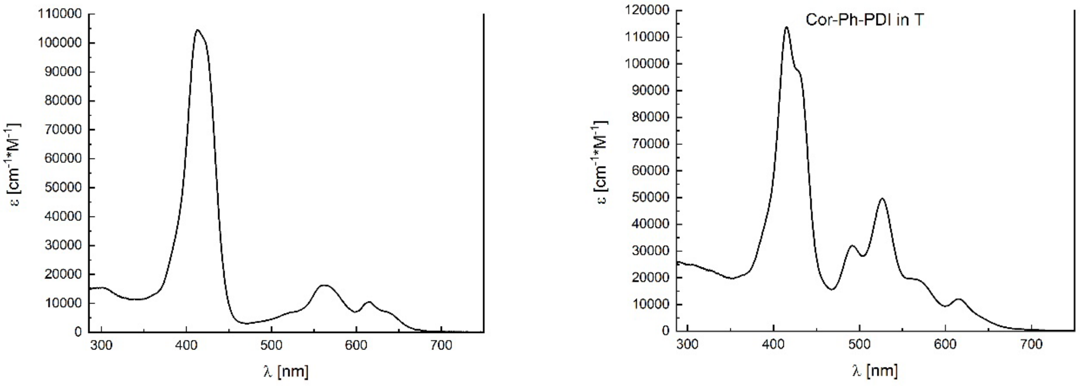

Figure S3. Molar absorption coefficient spectra (a) for **Cor-BiPh** dyad and (b) for **Cor-Ph-PDI** dyad in toluene.

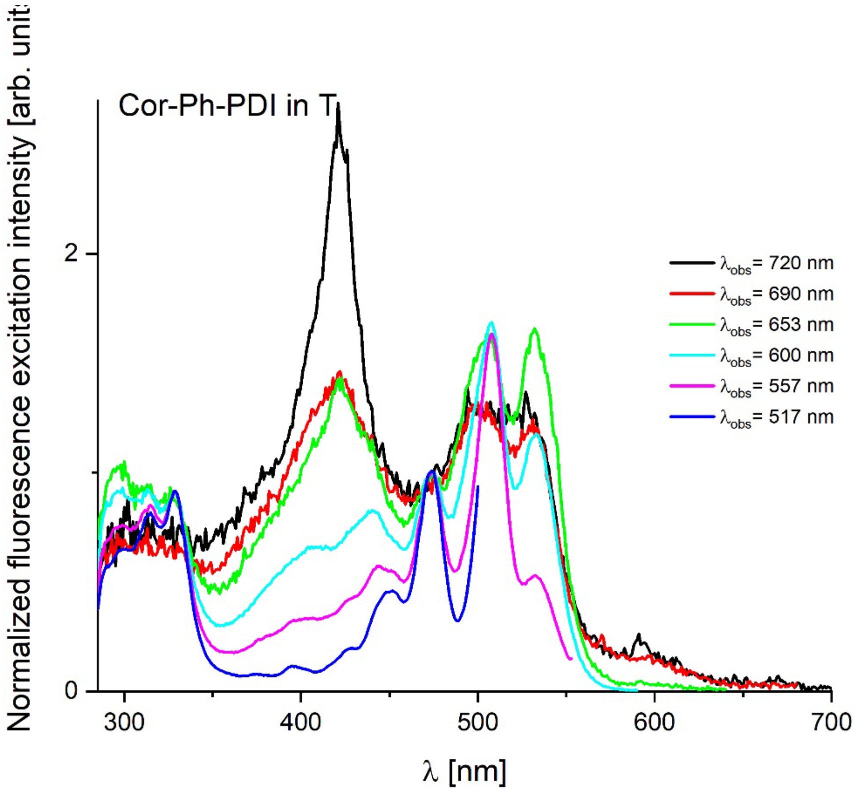

Figure S4. Fluorescence excitation spectra for **Cor-BiPh** dyad in toluene.

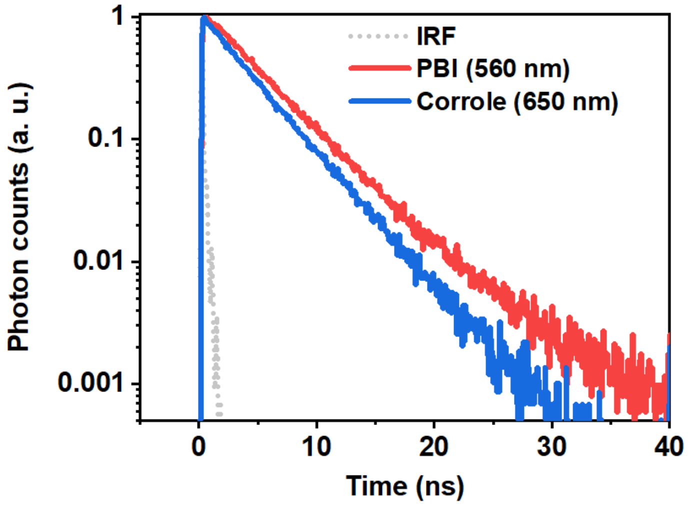

Figure S5. The emission lifetime of **PDI**, Corrole monomer in toluene, excited at 500 and 400 nm, the detected wavelength at 560 and 650 nm, respectively.

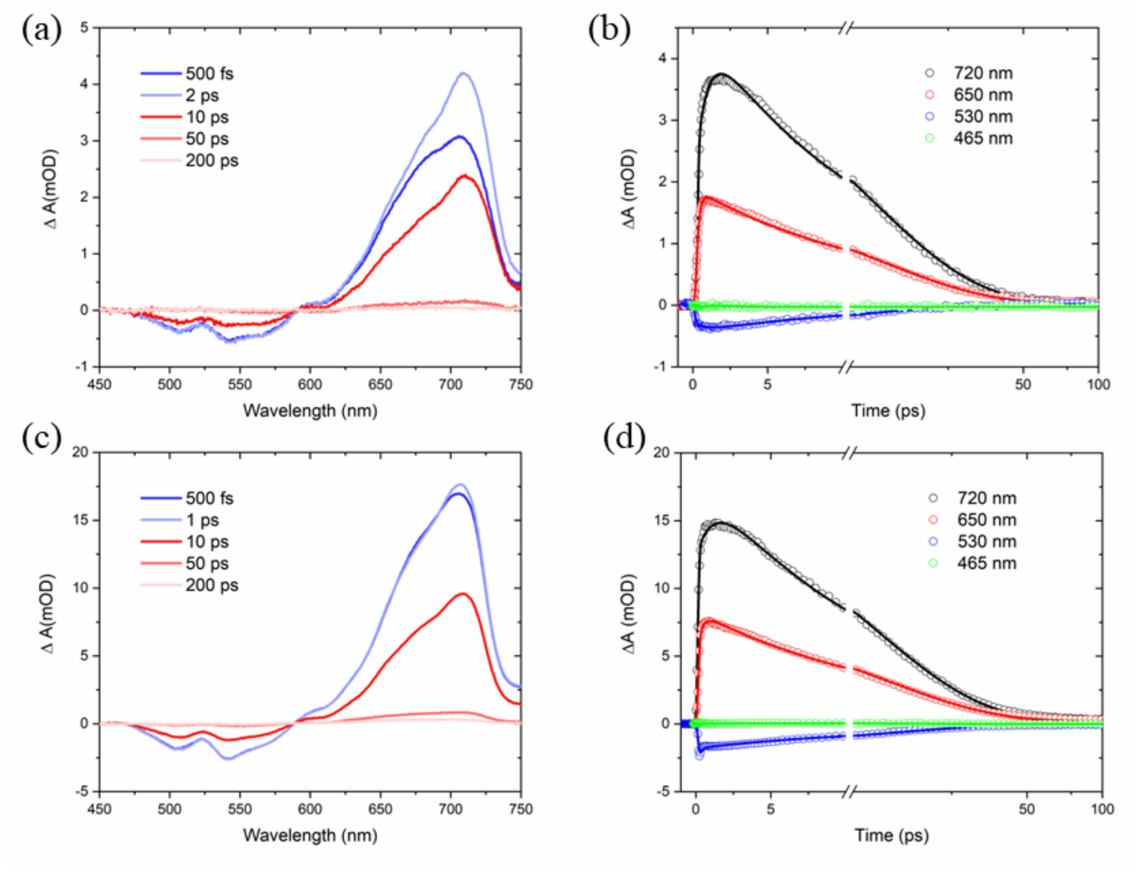

Figure S6. Transient absorption spectra (a) and kinetics (b) for **Cor-Ph-PDI** dyad, λ_{ex} = 400 nm in THF. Transient absorption spectra (c) and kinetics (d) for **Cor-Ph-PDI** dyad, λ_{ex} = 500 nm in THF.

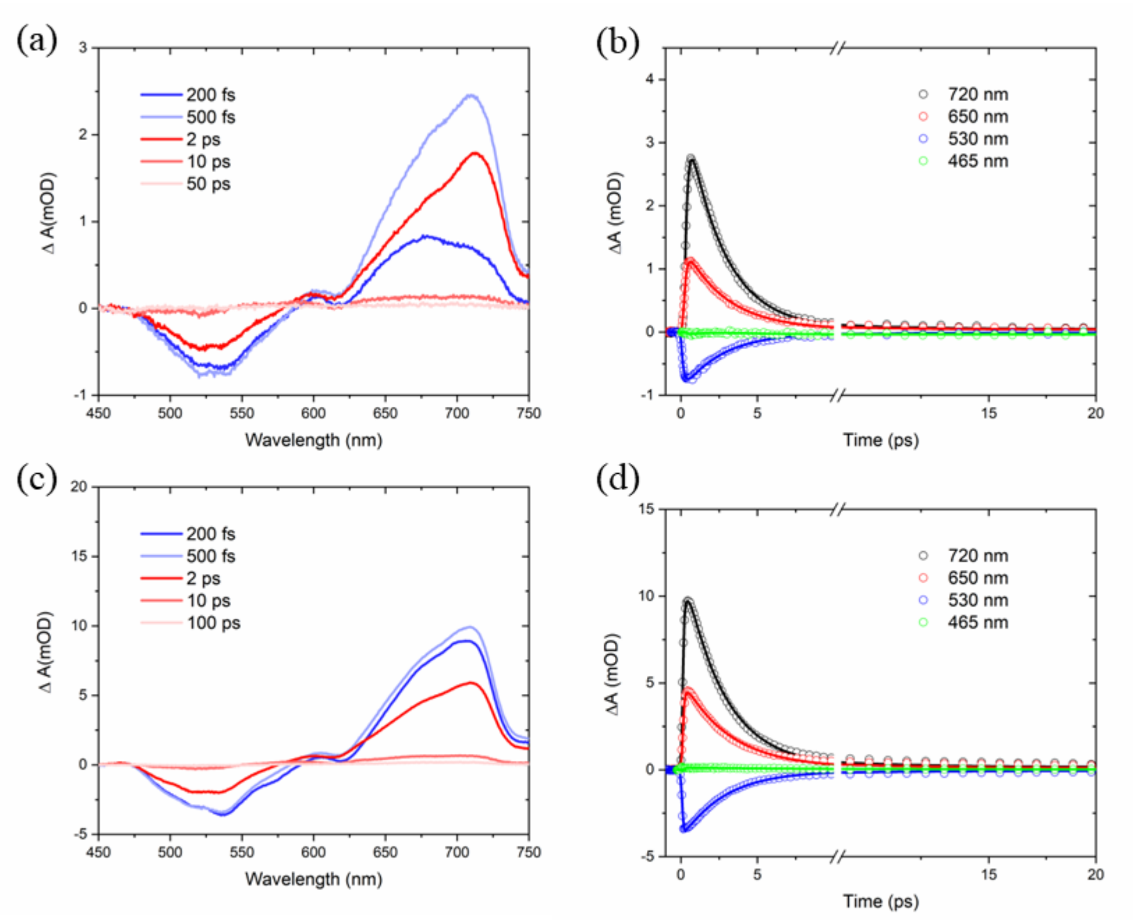

Figure S7. Transient absorption spectra (a) and kinetics (b) for **Cor-Ph-PDI** dyad, λ_{ex} = 400 nm in ACN. Transient absorption spectra (c) and kinetics (d) for **Cor-Ph-PDI** dyad, λ_{ex} = 500 nm in ACN.

5. Electrochemistry

Cyclic voltammograms were recorded using a Bio-Logic SP-50 potentiostat. Measurement conditions: A cylindrical three-electrode cell was equipped with a glassy carbon working electrode, a 25 mm platinum wire as the counter electrode and Ag/AgCl (3.0 M NaCl) electrode as the reference electrode. The scan rate for a typical experiment was 100 mV·s^{-1}. The solution of n-Bu_4NPF_6 (0.1 M) in dry dichloromethane was degassed by bubbling of the Ar gas before the measurement and cyclic voltammetry was carried out in an atmosphere of Ar gas at room temperature.

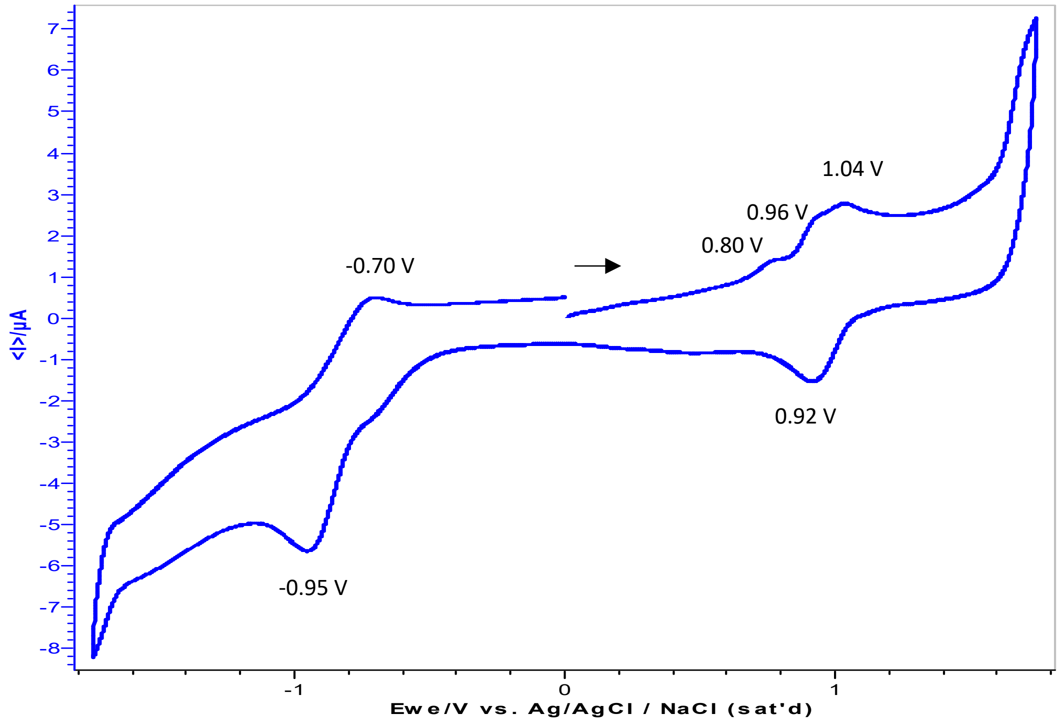

Figure S8. Cyclic voltammograms of **Cor-BiPh** in DCM , 0.1 M Bu_4NPF_6. The scan rate is 100 $mV.s^{-1}$

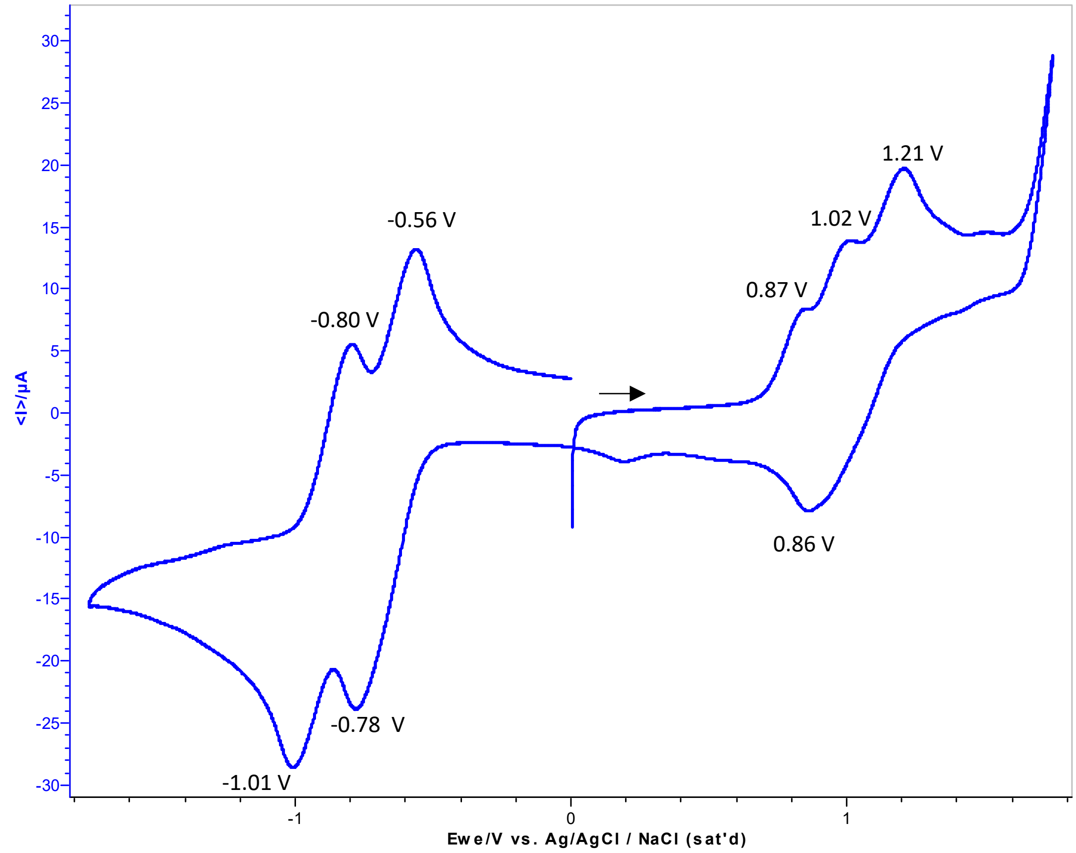

Figure S9. Cyclic voltammograms of **Cor-Ph-PDI** in DCM , 0.1 M Bu_4NPF_6. The scan rate is 100 $mV.s^{-1}$

6. ^{1}H and ^{13}C NMR, LRMS, HRMS spectral data

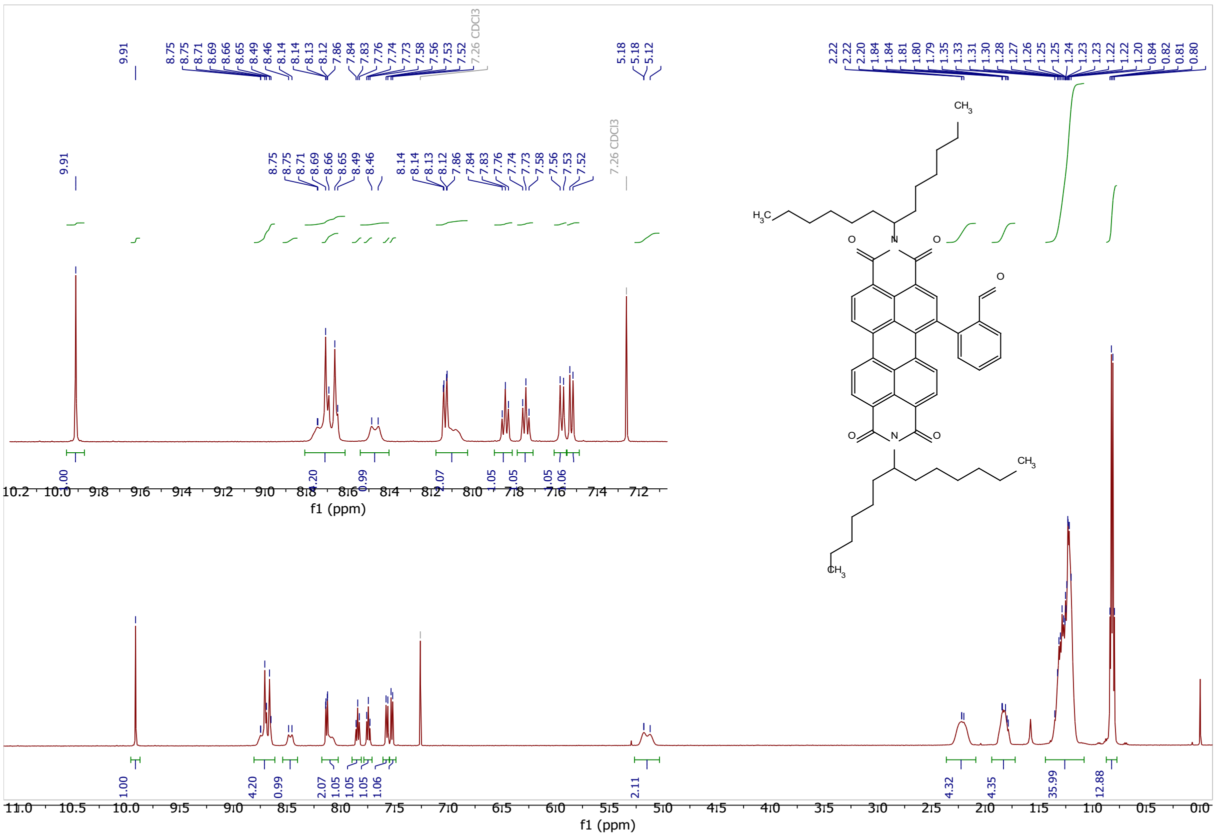

Figure S10. ^{1}H NMR spectrum of **PDI-PhCHO** (500 MHz, CD_2Cl_2)

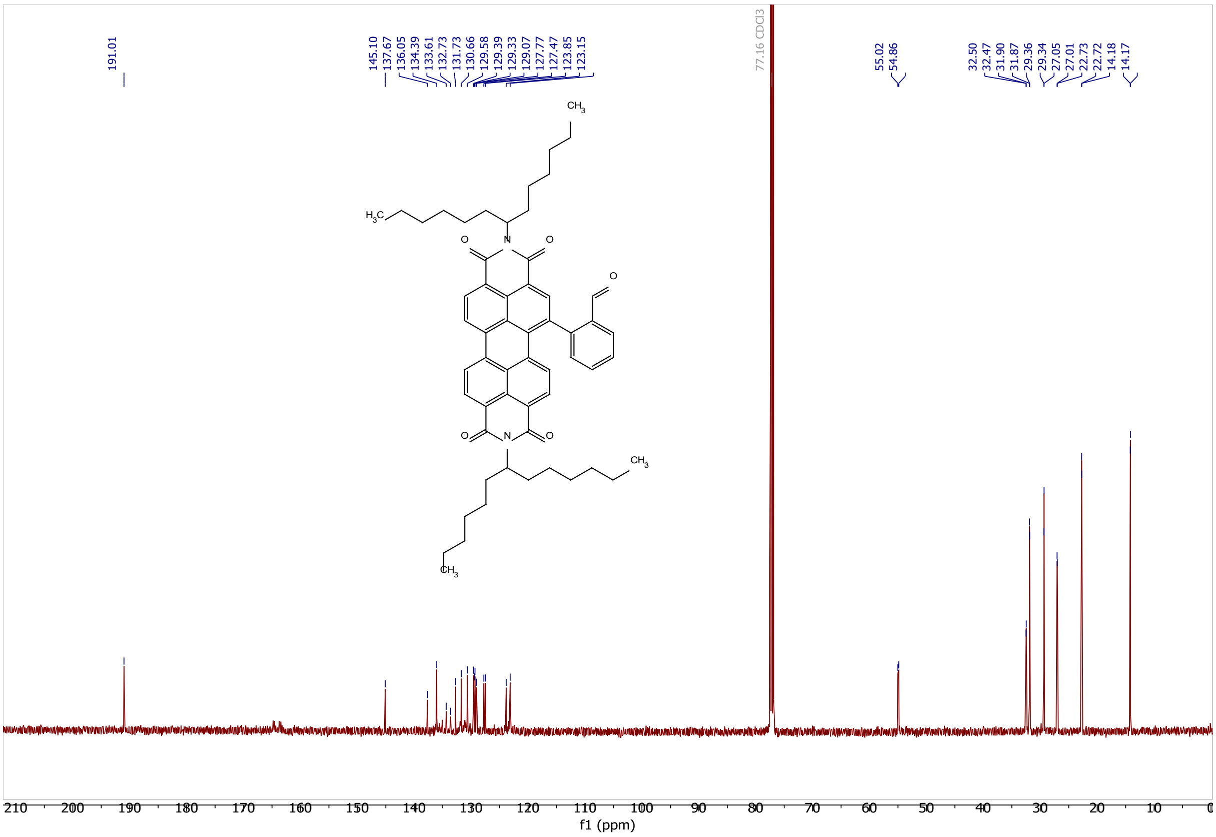

Figure S11. ^{13}C NMR spectrum of **PDI-PhCHO**(126 MHz, CD_2Cl_2

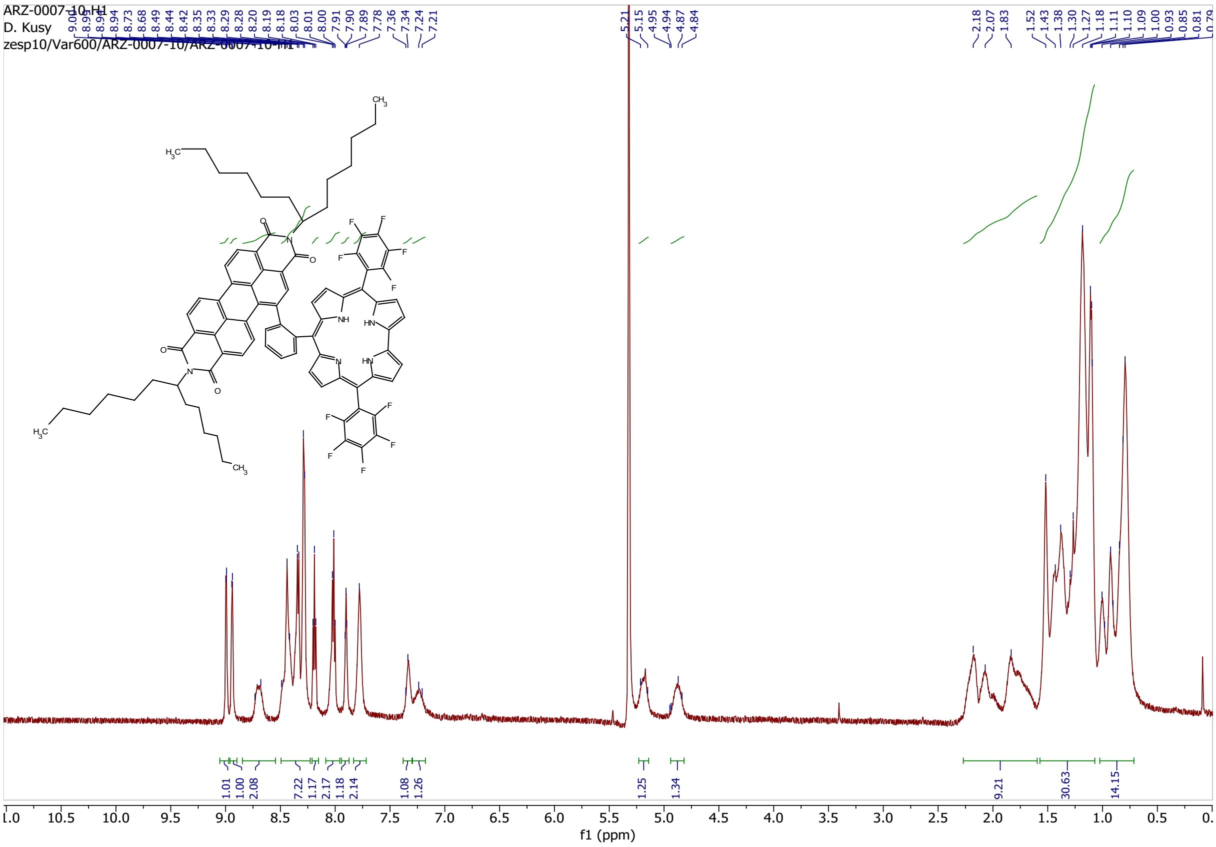

Figure S12. ^{1}H NMR spectrum of **Cor-Ph-PDI** (600 MHz, CD_2Cl_2)

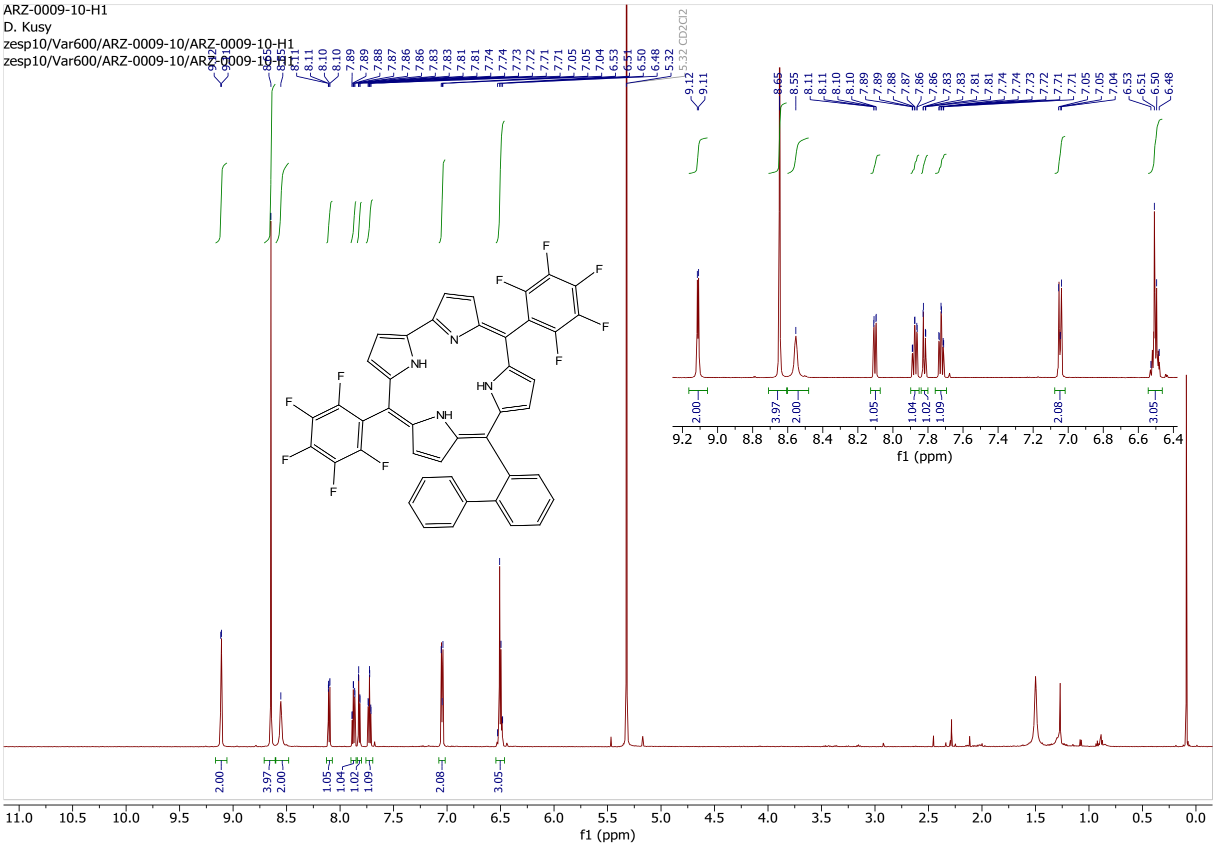

Figure S13. ^{1}H NMR spectrum of **Cor-BiPh** (600MHz, CD_2Cl_2)

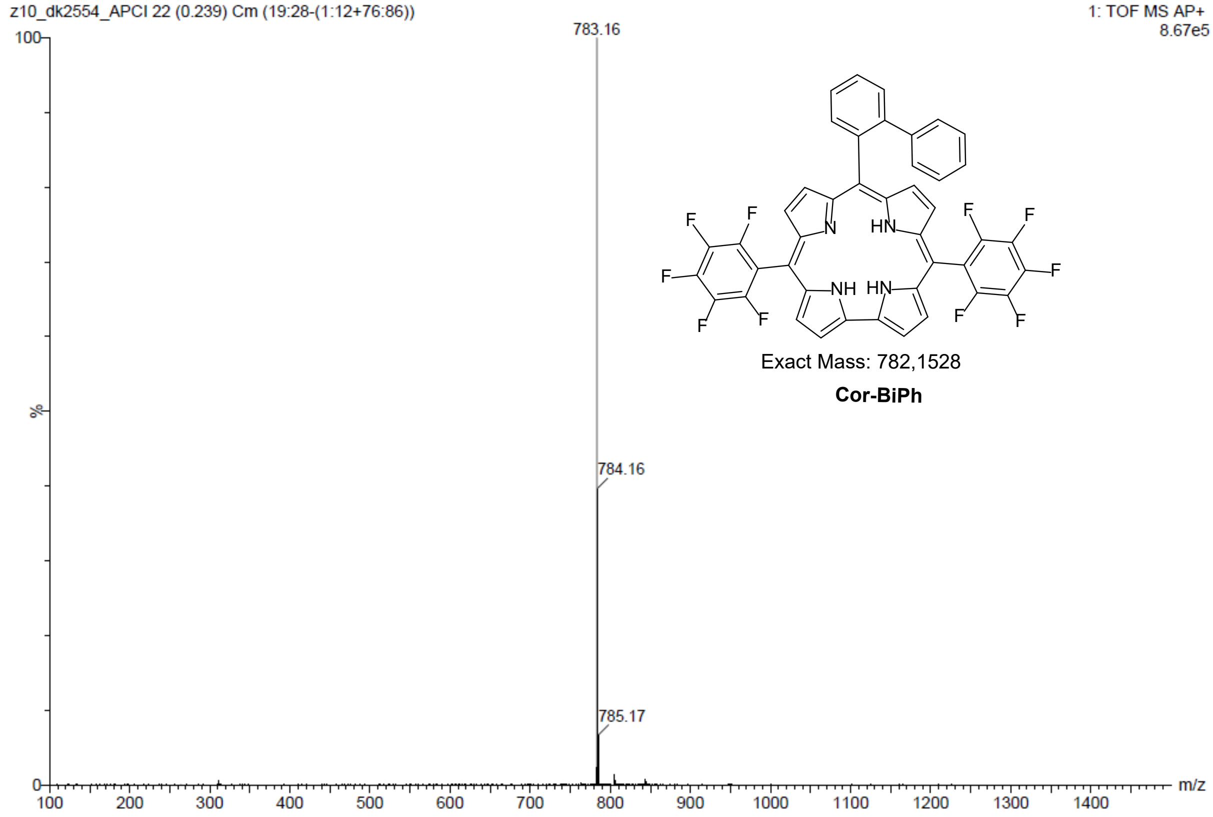

Figure S14. LRMS spectrum of **Cor-BiPh**

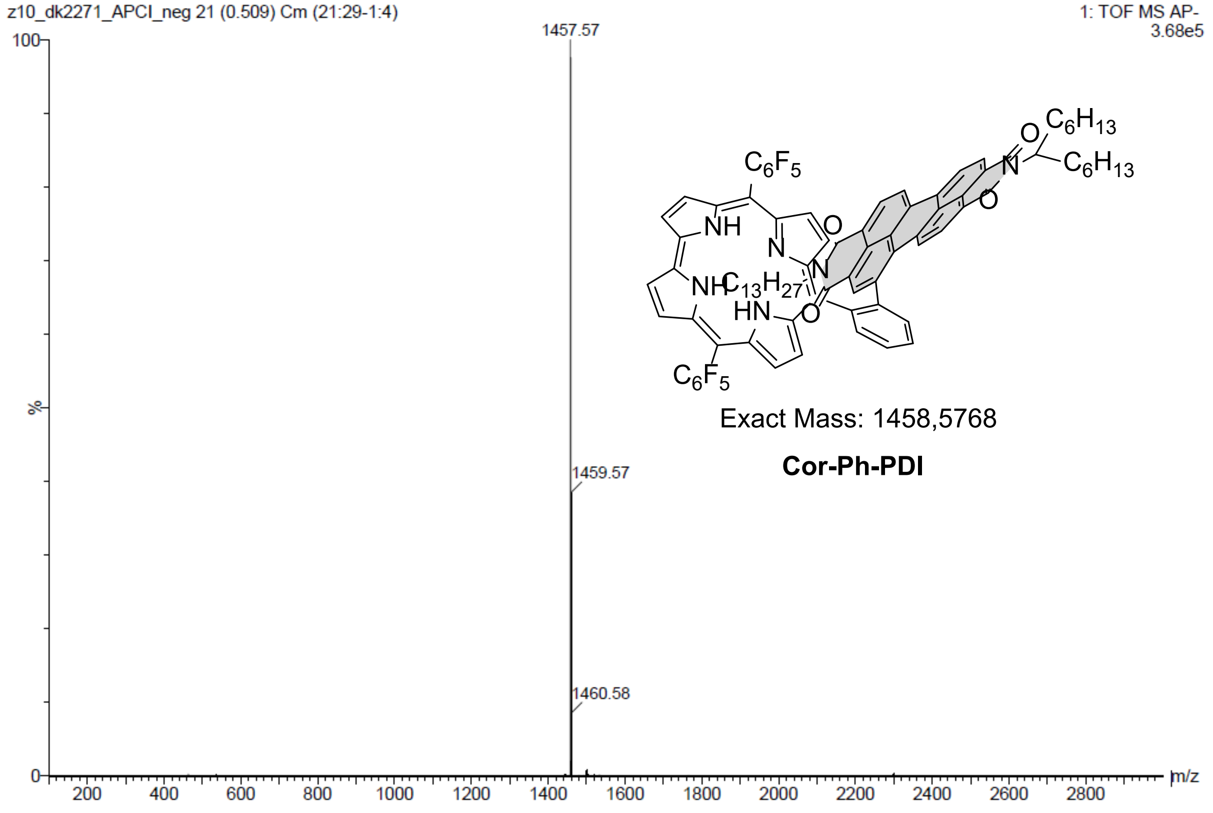

Single Mass Analysis
Tolerance = 3.0 mDa / DBE: min = -1.5, max = 300.0
Element prediction: Off
Number of isotope peaks used for i-FIT = 3
Monoisotopic Mass, Odd and Even Electron Ions
447 formula(e) evaluated with 2 results within limits (up to 50 closest results for each mass)
Elements Used:
C: 0-150 H: 0-200 N: 4-6 O: 3-4 F: 6-10

Mass	Calc. Mass	mDa	PPM	DBE	Formula	i-FIT	i-FIT Norm	Fit Conf %	C	H	N	O	F
1457.5685	1457.5690	-0.5	-0.3	48.5	C87 H75 N6 O4 F10	457.1	0.119	88.76	87	75	6	4	10
	1457.5679	0.6	0.4	52.5	C90 H74 N6 O3 F9	459.2	2.185	11.24	90	74	6	3	9

Figure S15. HRMS spectrum of **Cor-Ph-PDI**

7. References

(1) Holman, M. W.; Liu, R.; Adams, D. M. Single-Molecule Spectroscopy of Interfacial Electron Transfer. *J. Am. Chem. Soc.* **2003**, *125*, 12649–12654. https://doi.org/10.1021/ja0343104.

(2) Langhals, H.; Kirner, S. Novel Fluorescent Dyes by the Extension of the Core of Perylenetetracarboxylic Bisimides. *European J. Org. Chem.* **2000**, *2*, 365–380. https://doi.org/10.1002/(sici)1099-0690(200001)2000:2<365::aid-ejoc365>3.0.co;2-r.

(3) Rajasingh, P.; Cohen, R.; Shirman, E.; Shimon, L. J. W.; Rybtchinski, B. Selective Bromination of Perylene Diimides under Mild Conditions. *J. Org. Chem.* **2007**, *72*, 5973–5979. https://doi.org/10.1021/jo070367n.
