# Supplementary material for: Efficient Electron Transfer Driven by Excited-State Structural Relaxation in Corrole–Perylenedimiide Dyad
Source: J Phys Chem Lett. 2024 May 8;15(19):5231–8. doi: 10.1021/acs.jpclett.4c00916 (PMC11103693; doi:10.1021/acs.jpclett.4c00916)
Supplement: Supplementary file 2 — jz4c00916_si_002.pdf [file jz4c00916_si_002.pdf]

Cartesian Coordinates

Dyad:

Ground state optimization - MP2/def2-SVP				Ground state optimization - PW6B95-D3/6-31G(d,p)			
N	-4.20697722	2.15268866	-1.40974675	N	-4.11700	1.67445	-0.95833
N	-2.00478750	3.54463038	-1.74838227	N	-1.84495	2.90350	-0.95463
N	-0.51288045	1.19122668	-2.03760217	N	-0.47570	0.70421	-1.77957
N	-2.95913820	-0.23182485	-1.44483878	N	-3.03639	-0.64862	-1.56638
C	-4.38330166	3.47573283	-1.58501873	C	-4.18294	3.01503	-0.75599
C	-5.37085414	1.51602641	-1.15891584	C	-5.31796	1.07325	-0.78110
C	0.47941005	2.13234437	-2.29711940	C	0.58387	1.59279	-1.71291
C	-0.00244071	-0.07303980	-1.87764535	C	-0.03425	-0.58825	-1.93091
C	-3.18291916	4.20287675	-1.97539865	C	-2.95601	3.70999	-0.86245
C	-0.98041389	4.12247675	-2.43544954	C	-0.71841	3.63284	-1.24259
C	-4.24971448	-0.69737056	-1.18198078	C	-4.29249	-1.11736	-1.26464
C	-2.15747629	-1.25717450	-1.45067228	C	-2.23968	-1.73658	-1.68506
C	-5.73444237	3.73157167	-1.40707404	C	-5.52881	3.30251	-0.43533
C	-6.35830037	2.49346560	-1.14013790	C	-6.22893	2.10733	-0.44861
C	1.72566787	1.36776963	-2.27371253	C	1.75830	0.79689	-1.88203
C	1.44635912	0.08242424	-2.03665141	C	1.38497	-0.50664	-2.02626
C	-2.89596685	5.26828496	-2.79042791	C	-2.51096	5.01804	-1.08403
C	-1.50269271	5.22205918	-3.07745347	C	-1.13623	4.97443	-1.31017
C	-4.18722605	-2.14921055	-1.04616432	C	-4.28254	-2.55993	-1.22815
C	-2.90719395	-2.50231893	-1.21395439	C	-3.01112	-2.94757	-1.47879
C	-5.36718774	0.07575598	-1.04062918	C	-5.40029	-0.32417	-0.92348
C	-0.70281310	-1.21455436	-1.62157560	C	-0.83128	-1.72646	-1.85942
C	0.32115370	3.46402412	-2.49792536	C	0.50430	2.96515	-1.47933
H	-6.21300277	4.69574734	-1.45915164	H	-5.91929	4.27657	-0.19545
H	-7.40956717	2.33768293	-0.95926418	H	-7.27601	1.97291	-0.23588
H	2.70499785	1.79480424	-2.41213163	H	2.76306	1.18292	-1.88073
H	2.15666706	-0.71856535	-1.96193782	H	2.03374	-1.34928	-2.17651
H	-3.61283027	5.95320710	-3.21351524	H	-3.14011	5.88949	-1.14712
H	-0.96933382	5.88072455	-3.74376604	H	-0.51029	5.80704	-1.57996
H	-5.01806088	-2.80178475	-0.83443780	H	-5.12507	-3.18991	-0.99743
H	-2.49423037	-3.49557773	-1.17360567	H	-2.62536	-3.95206	-1.50343
H	-3.37552756	1.61787900	-1.55014997	H	-3.34322	1.09059	-1.27925
H	-1.86257283	2.91680693	-0.98579173	H	-1.78455	2.03499	-0.44611
H	-1.47608410	1.31585863	-2.25263390	H	-1.43804	0.93066	-1.95910
C	0.00526139	-2.54891955	-1.53774262	C	-0.13771	-3.03835	-1.68607
C	0.60672418	-3.04952331	-0.37032265	C	0.55314	-3.31419	-0.48794
C	-0.02896155	-3.37065801	-2.66973716	C	-0.25182	-4.04601	-2.64465
C	1.06023996	-4.37196717	-0.35164209	C	1.03380	-4.60316	-0.25583
C	0.46281946	-4.66398700	-2.65071971	C	0.26598	-5.31244	-2.41806
C	0.99073378	-5.17676971	-1.47318512	C	0.89425	-5.59720	-1.21097
H	-0.47423743	-2.98305216	-3.57633913	H	-0.77264	-3.82613	-3.56708
H	1.49220362	-4.76293319	0.55972016	H	1.53938	-4.81153	0.67810
H	0.41650368	-5.27376614	-3.54320997	H	0.16594	-6.07925	-3.17409
H	1.35275332	-6.19543512	-1.43117564	H	1.27650	-6.58975	-1.01557
C	-0.51737305	-1.97035448	1.52252007	C	-0.58438	-1.84805	1.05736
C	0.72681607	-2.24649247	0.89959814	C	0.66667	-2.25274	0.55380
C	1.91187444	-1.85015063	1.51760937	C	1.84307	-1.62622	0.99866
C	1.82415501	-1.20986246	2.81041113	C	1.71525	-0.58569	1.98137
C	0.56245409	-0.90557590	3.36617675	C	0.43025	-0.14758	2.39609
C	-0.61364580	-1.30363260	2.70123844	C	-0.72048	-0.79747	1.92073
C	2.97913643	-0.87328757	3.58219275	C	2.84332	0.06386	2.55437
C	0.44202332	-0.22279576	4.59387931	C	0.27604	0.92477	3.29041
C	2.82737760	-0.18718300	4.76796701	C	2.65316	1.14365	3.40876
C	1.56650743	0.14883315	5.27514299	C	1.38607	1.57864	3.77543
C	-0.89569921	0.10570842	5.16481061	C	-1.06144	1.37031	3.72748
C	-1.96156029	-1.03686998	3.28303056	C	-2.07750	-0.35681	2.30425
N	-2.02091177	-0.32705076	4.47116562	N	-2.16684	0.66450	3.24481
O	-0.98262956	0.71313703	6.18538748	O	-1.19466	2.31603	4.48435
O	-2.94683608	-1.42199701	2.73613629	O	-3.07201	-0.86377	1.81411
H	-1.42743859	-2.30270988	1.05198313	H	-1.48045	-2.35510	0.73011

H	1.47254981	0.68537097	6.20718439
H	3.69121524	0.10974745	5.33881039
C	4.31592935	-1.27841990	3.10148887
C	5.44463336	-1.15667594	3.88223484
C	6.71775738	-1.47882817	3.39202571
C	6.86665423	-1.91113569	2.10379460
C	5.72992555	-2.06767212	1.28243269
C	4.43368054	-1.79522957	1.77990794
C	3.28841148	-2.02932294	0.94501007
C	3.53067131	-2.40808372	-0.35761968
C	4.82525862	-2.63266725	-0.85743561
C	5.91411077	-2.49214506	-0.05025628
H	5.37368578	-0.80688429	4.89865183
H	7.58941535	-1.37472230	4.02052986
H	4.96084177	-2.93787209	-1.88435317
H	2.72247270	-2.56829606	-1.03984773
C	-3.35128515	-0.00840208	5.06251692
H	-3.10281721	0.54824979	5.95575361
C	-4.10009491	-1.26436925	5.50169501
C	-4.17306885	0.91250149	4.16431223
H	-3.59526598	1.79835993	3.89406609
H	-4.50587652	0.41886349	3.25556473
H	-5.05411688	1.24855830	4.71392805
H	-4.42377907	-1.86745762	4.65723696
H	-4.98213105	-0.96949747	6.07300581
H	-3.47342900	-1.87644566	6.15311209
C	7.27460423	-2.77233620	-0.59036563
C	8.23328812	-2.21464542	1.59157817
N	8.35269455	-2.62695563	0.26738936
O	9.18374796	-2.10167078	2.30012119
O	7.41956591	-3.11206195	-1.72313092
C	9.72476636	-2.91640228	-0.23529246
H	10.35279822	-2.73312014	0.62581550
C	10.15390118	-1.94071905	-1.32852800
C	9.89857174	-4.38337073	-0.62137572
H	9.59082008	-5.03437560	0.19898556
H	9.33148890	-4.64668906	-1.51059377
H	10.95520278	-4.57513745	-0.81675090
H	9.59661235	-2.08358275	-2.25090464
H	11.21454717	-2.08865758	-1.53962764
H	10.02435727	-0.90974559	-0.99415300
C	1.49354143	4.31516322	-2.85923526
C	2.14654355	4.19711058	-4.07936730
C	1.94949548	5.30215227	-1.99063942
C	3.20857415	5.01489468	-4.42157210
C	3.00655950	6.12852263	-2.31485140
C	3.63751026	5.98344362	-3.53674722
C	-6.68921129	-0.57234563	-0.76644213
C	-7.28176355	-0.48834360	0.48685134
C	-7.38728133	-1.24872660	-1.75761580
C	-8.50979090	-1.06413273	0.75037300
C	-8.61766856	-1.82938823	-1.51283017
C	-9.17956617	-1.73490609	-0.25482517
F	-6.88695130	-1.35559344	-2.96225721
F	-9.25289387	-2.46351789	-2.46642431
F	-10.34072790	-2.28028754	-0.01506344
F	-9.04158469	-0.97678444	1.94358144
F	-6.67626852	0.14196879	1.45977494
F	1.38080826	5.45816161	-0.82280409
F	3.41937633	7.04209989	-1.47278840
F	4.63893372	6.75908319	-3.85028200
F	3.80320281	4.87771652	-5.58028210
F	1.76652668	3.29415894	-4.94882612

H	1.24630	2.41518	4.44470
H	3.50699	1.67956	3.79289
C	4.18178	-0.40632	2.22453
C	5.31042	0.05076	2.89273
C	6.59188	-0.34289	2.52036
C	6.76624	-1.19926	1.45708
C	5.64576	-1.70631	0.77395
C	4.32897	-1.34829	1.17329
C	3.20347	-1.92276	0.50489
C	3.47099	-2.73257	-0.59573
C	4.76548	-3.04578	-1.00644
C	5.85248	-2.56501	-0.31893
H	5.20717	0.72517	3.72854
H	7.46609	0.01973	3.04170
H	4.93336	-3.68286	-1.86318
H	2.66729	-3.15389	-1.16763
C	-3.50327	1.11917	3.70084
H	-3.26894	1.80955	4.50582
C	-4.33916	-0.02053	4.26700
C	-4.21182	1.87968	2.59200
H	-3.60806	2.72169	2.25291
H	-4.41856	1.22012	1.75513
H	-5.16045	2.27062	2.95941
H	-4.70774	-0.67611	3.48378
H	-5.19193	0.40603	4.79536
H	-3.76327	-0.60840	4.98167
C	7.20996	-2.94381	-0.75646
C	8.13819	-1.57048	1.05967
N	8.28052	-2.42556	-0.03678
O	9.10724	-1.14490	1.66331
O	7.38261	-3.68224	-1.71164
C	9.66104	-2.78559	-0.43993
H	10.27694	-2.25368	0.27930
C	9.98653	-2.26409	-1.83278
C	9.91520	-4.27816	-0.28155
H	9.66953	-4.60529	0.72849
H	9.33347	-4.85706	-0.99382
H	10.97331	-4.47832	-0.45049
H	9.40634	-2.77851	-2.59419
H	11.04595	-2.42387	-2.03359
H	9.79105	-1.19400	-1.89842
C	1.76460	3.73274	-1.39532
C	2.07975	4.76038	-2.27735
C	2.68538	3.46772	-0.38312
C	3.24980	5.49420	-2.16405
C	3.86789	4.17618	-0.25849
C	4.14910	5.19700	-1.15264
C	-6.69353	-0.96916	-0.62474
C	-7.30844	-0.81912	0.61692
C	-7.36124	-1.74934	-1.56623
C	-8.52650	-1.40909	0.91053
C	-8.57608	-2.35507	-1.29138
C	-9.16250	-2.18070	-0.04826
F	-6.83588	-1.93060	-2.77570
F	-9.18781	-3.08975	-2.21485
F	-10.32818	-2.75287	0.22369
F	-9.08112	-1.24971	2.10797
F	-6.72346	-0.10422	1.57778
F	2.44004	2.50539	0.50624
F	4.72379	3.89480	0.71900
F	5.27520	5.88843	-1.03978
F	3.51935	6.46952	-3.02516
F	1.24154	5.06941	-3.26690

First excited state optimization - PW6B95-D3/6-31G(d,p):

N	0.867336000	2.434385000	-0.890217000
N	-1.737706000	2.255071000	-0.797304000
N	-1.690394000	0.531994000	1.323595000
N	1.192256000	0.710055000	1.038545000
C	0.184912000	3.309629000	-1.672282000
C	2.182235000	2.734755000	-0.843918000
C	-3.044856000	0.619208000	1.151309000
C	-1.349133000	-0.633288000	1.969756000
C	-1.223697000	3.293359000	-1.513973000
C	-3.035842000	2.516797000	-0.415128000
C	2.510870000	1.133698000	1.012532000
C	1.132250000	-0.272746000	1.944987000
C	1.141522000	4.169507000	-2.249057000
C	2.381104000	3.808160000	-1.743374000
C	-3.601744000	-0.542744000	1.741044000
C	-2.571506000	-1.317102000	2.211971000
C	-2.253158000	4.252165000	-1.655274000
C	-3.363321000	3.777667000	-0.990629000
C	3.270260000	0.407834000	2.004784000
C	2.421018000	-0.480636000	2.568862000
C	3.002160000	2.102044000	0.134723000
C	-0.042680000	-1.028667000	2.271386000
C	-3.704125000	1.685886000	0.470001000
H	0.933945000	4.966842000	-2.941571000
H	3.325671000	4.275297000	-1.961927000
H	-4.644573000	-0.802169000	1.730647000
H	-2.644129000	-2.301369000	2.636860000
H	-2.147455000	5.212064000	-2.130412000
H	-4.296402000	4.294863000	-0.849557000
H	4.315721000	0.537103000	2.222223000
H	2.630067000	-1.207468000	3.334917000
H	0.520950000	1.704950000	-0.261769000
H	-1.409359000	1.305249000	-0.931424000
H	-1.017563000	1.261869000	1.156101000
C	0.146049000	-2.321632000	2.962505000
C	0.982190000	-3.324519000	2.411802000
C	-0.489750000	-2.545569000	4.186202000
C	1.173410000	-4.495207000	3.145871000
C	-0.292276000	-3.722478000	4.890522000
C	0.549510000	-4.695874000	4.368261000
H	-1.112508000	-1.763379000	4.600319000
H	1.807081000	-5.265529000	2.726910000
H	-0.780635000	-3.871437000	5.843285000
H	0.714275000	-5.618170000	4.908675000
C	3.061227000	-3.254775000	1.106543000
C	1.678572000	-3.116703000	1.123056000
C	1.017111000	-2.591266000	-0.022650000
C	1.790376000	-1.894882000	-0.989260000
C	3.207574000	-1.999083000	-0.950456000
C	3.824373000	-2.760236000	0.060391000
C	1.178907000	-1.070382000	-1.974185000
C	3.989054000	-1.359807000	-1.923748000
C	2.004200000	-0.406245000	-2.900424000
C	3.375131000	-0.560405000	-2.884278000
C	5.441107000	-1.528435000	-1.944738000
C	5.274374000	-2.935864000	0.083817000
N	6.000910000	-2.338687000	-0.945523000
O	6.143700000	-0.991371000	-2.790938000
O	5.833543000	-3.585162000	0.960769000
H	3.583895000	-3.686913000	1.950198000
H	4.004254000	-0.079257000	-3.619890000
H	1.558289000	0.202990000	-3.672744000

C	-0.258178000	-0.970481000	-1.999193000
C	-0.953039000	-0.077864000	-2.843163000
C	-2.330030000	-0.121516000	-2.961756000
C	-3.074677000	-1.078447000	-2.275690000
C	-2.421865000	-1.978816000	-1.411808000
C	-1.014141000	-1.884000000	-1.211764000
C	-0.390494000	-2.744268000	-0.267064000
C	-1.163613000	-3.750663000	0.346676000
C	-2.509070000	-3.884746000	0.078685000
C	-3.157077000	-2.991575000	-0.772330000
H	-0.400007000	0.646042000	-3.425164000
H	-2.860234000	0.559023000	-3.613820000
H	-3.092050000	-4.687590000	0.507956000
H	-0.678516000	-4.467565000	0.990905000
C	7.466156000	-2.534166000	-0.986737000
H	7.768738000	-1.962513000	-1.859415000
C	7.821913000	-3.996235000	-1.218499000
C	8.152909000	-1.940458000	0.235978000
H	7.844860000	-0.906970000	0.380176000
H	7.922106000	-2.509770000	1.131946000
H	9.232238000	-1.950771000	0.079551000
H	7.538094000	-4.608349000	-0.366471000
H	8.898170000	-4.084673000	-1.370190000
H	7.324248000	-4.375779000	-2.110693000
C	-4.574076000	-3.184354000	-1.068474000
C	-4.511835000	-1.178938000	-2.514141000
N	-5.171091000	-2.279298000	-1.947224000
O	-5.115384000	-0.362427000	-3.200980000
O	-5.216429000	-4.111027000	-0.585737000
C	-6.562032000	-2.526881000	-2.391532000
H	-6.777573000	-1.668418000	-3.021425000
C	-7.577261000	-2.553953000	-1.256683000
C	-6.619132000	-3.788560000	-3.244194000
H	-5.916508000	-3.724887000	-4.074788000
H	-6.385345000	-4.667658000	-2.647831000
H	-7.621571000	-3.904534000	-3.657149000
H	-7.433603000	-3.420480000	-0.618744000
H	-8.577191000	-2.598304000	-1.690641000
H	-7.519364000	-1.652545000	-0.653340000
C	-5.125170000	1.966377000	0.768356000
C	-5.494077000	3.139355000	1.421277000
C	-6.144049000	1.095252000	0.402755000
C	-6.817148000	3.437446000	1.699054000
C	-7.473917000	1.366391000	0.678375000
C	-7.809943000	2.544771000	1.326713000
C	4.386956000	2.604086000	0.266522000
C	5.513020000	1.790132000	0.163950000
C	4.614469000	3.961229000	0.503395000
C	6.797753000	2.301317000	0.256019000
C	5.888536000	4.491344000	0.600761000
C	6.986478000	3.656432000	0.472264000
F	3.587709000	4.794648000	0.668470000
F	6.060635000	5.787119000	0.832245000
F	8.210829000	4.149756000	0.566313000
F	7.851480000	1.504830000	0.142904000
F	5.380466000	0.482050000	-0.018044000
F	-5.841910000	-0.040882000	-0.217357000
F	-8.426141000	0.515278000	0.322926000
F	-9.077806000	2.816612000	1.591759000
F	-7.136069000	4.564151000	2.323539000
F	-4.561336000	4.015687000	1.795250000

Corrole:

Ground state optimization - MP2/def2-SVP			
N	-4.26011381	2.12478303	-1.46230523
N	-2.03277868	3.49046700	-1.75957645
N	-0.57333444	1.12787865	-2.05152531
N	-3.05344113	-0.27530469	-1.48946116
C	-4.41411562	3.45216514	-1.62343899
C	-5.43286746	1.50611890	-1.20483332
C	0.44249931	2.05169070	-2.27000491
C	-0.08993027	-0.14488614	-1.87755858
C	-3.19959399	4.16472044	-1.99894287
C	-0.99136183	4.05802675	-2.42879623
C	-4.34720934	-0.72423810	-1.22128676
C	-2.25979437	-1.30686608	-1.47732911
C	-5.75987426	3.72963524	-1.43495461
C	-6.40330166	2.50042359	-1.17281171
C	1.67585175	1.26560493	-2.20486148
C	1.36792395	-0.01580765	-1.98110983
C	-2.88782631	5.22918433	-2.80576589
C	-1.49099533	5.16640629	-3.07383532
C	-4.29878148	-2.17606380	-1.06437586
C	-3.01940851	-2.54177690	-1.21792285
C	-5.45209817	0.06662675	-1.08231259
C	-0.80967039	-1.27750733	-1.64344691
C	0.30647498	3.38702159	-2.46478065
H	-6.22161153	4.70257048	-1.47447025
H	-7.45576613	2.36116353	-0.98494797
H	2.66560250	1.67987054	-2.30234810
H	2.05573252	-0.83655931	-1.87285687
H	-3.58998958	5.92514651	-3.23556388
H	-0.93976784	5.82168362	-3.72889122
H	-5.13847470	-2.81807156	-0.85283272
H	-2.60881096	-3.53568006	-1.16190643
H	-3.43743788	1.57730274	-1.60847104
H	-1.90944883	2.85233660	-1.00211972
H	-1.52411311	1.27138626	-2.30537254
C	-0.06952495	-2.58821672	-1.54412608
C	0.60040533	-3.00207549	-0.38053016
C	-0.03762629	-3.41404261	-2.66976492
C	1.27691362	-4.22583288	-0.39363257
C	0.63082871	-4.62711949	-2.66518750
C	1.29313840	-5.03500553	-1.51705063
H	-0.54535757	-3.08770103	-3.56800286
H	1.78679290	-4.54966659	0.50370950
H	0.63713385	-5.24569876	-3.55297258
H	1.81900758	-5.98051611	-1.49370919
C	-0.50778735	-1.84430449	1.57839325
C	0.64869170	-2.18420856	0.87652579
C	1.87940060	-1.78209817	1.39577839
C	1.95374573	-1.05205803	2.57434617
C	0.79576444	-0.71608185	3.25915905
C	-0.43489452	-1.11784224	2.75782989
H	-1.47338132	-2.15991277	1.21052729
C	1.49794601	4.22779624	-2.78296050
C	2.19657844	4.10183432	-3.97691604
C	1.93061862	5.21206626	-1.89901194
C	3.27932265	4.90830366	-4.27892045
C	3.00719548	6.02769947	-2.18374122
C	3.68353376	5.87423359	-3.37997972
C	-6.78256628	-0.55875530	-0.79493185
C	-7.30998533	-0.56079090	0.48932886
C	-7.55034997	-1.12519546	-1.80261310
C	-8.54584655	-1.11324387	0.76599219
C	-8.78963525	-1.68131650	-1.54497906
C	-9.28735079	-1.67337997	-0.25664799
F	-7.10925060	-1.14764723	-3.03413525
F	-9.49300536	-2.21181773	-2.51374345
F	-10.45577849	-2.19736825	-0.00443431
F	-9.01669976	-1.10966413	1.98785094
F	-6.63032709	-0.04031699	1.47891163
F	1.32028111	5.37603006	-0.75352024
F	3.39665130	6.93879392	-1.32746268

F	4.70439110	6.63953655	-3.65582643
F	3.91699323	4.76297239	-5.41392717
F	1.84171753	3.20219092	-4.86038619
H	-1.34463135	-0.86871466	3.28841397
H	0.85076287	-0.14776261	4.17841291
H	2.91927205	-0.74558708	2.95547553
H	2.79001735	-2.03684492	0.86904296

PDI:

Ground state optimization - MP2/def2-SVP			
C	-0.51078186	-1.72189648	1.21041418
C	0.75721498	-2.08769900	0.72979143
C	1.90532590	-1.82995632	1.44419056
C	1.79546198	-1.17212155	2.70793304
C	0.51493979	-0.80927668	3.18050618
C	-0.63767314	-1.09196140	2.41659050
C	2.93955616	-0.86943425	3.50889796
C	0.36820953	-0.15839838	4.42412415
C	2.75370183	-0.23369146	4.71566243
C	1.47494848	0.12201806	5.17501234
C	-0.97949611	0.22759757	4.92983803
C	-1.99321738	-0.71314348	2.90670546
N	-2.07885349	-0.07332233	4.13987667
O	-1.09984619	0.77690002	5.98014841
O	-2.96138815	-0.95487797	2.25669675
H	-1.39606305	-1.93561123	0.63034268
H	1.35595229	0.62026499	6.12556572
H	3.59433667	0.00932735	5.34432750
C	4.28471625	-1.25031387	3.01228811
C	5.43289011	-0.99204266	3.72641731
C	6.70091937	-1.35762739	3.24560906
C	6.82741929	-1.98773701	2.03966837
C	5.67502809	-2.27104479	1.27578181
C	4.39451214	-1.90836602	1.74854344
C	3.25042845	-2.21118230	0.94775359
C	3.43626361	-2.84720910	-0.25884639
C	4.71493842	-3.20302078	-0.71819272
C	5.82206872	-2.92238027	0.03233171
H	5.38619635	-0.49748267	4.68254229
H	7.58648417	-1.14366732	3.82522627
H	4.83350374	-3.70143455	-1.66866822
H	2.59568658	-3.09029938	-0.88755533
C	7.16898113	-3.30916617	-0.47507183
C	8.18208578	-2.36722425	1.54781336
N	8.27048459	-3.00525902	0.31972623
O	9.15290802	-2.12656457	2.19509301
O	7.28548804	-3.85856026	-1.52525925
H	0.80385747	-2.58203636	-0.22644816
C	-3.43910128	0.30031384	4.61901554
H	-4.08915956	-0.04460005	3.82658004
C	9.61137017	-3.39691854	-0.19804928
H	9.38846358	-3.86320283	-1.14798831
C	-3.61585328	1.81311007	4.72645354
H	-3.02546487	2.24172929	5.53226950
H	-3.33819829	2.29864529	3.78908239
H	-4.66814454	2.03435835	4.91411209
C	-3.82942983	-0.45346832	5.88818819
H	-4.88574135	-0.27348813	6.09627779
H	-3.69822583	-1.52837972	5.75088407
H	-3.25009202	-0.13640305	6.75157115
C	10.28190376	-4.45090189	0.67991382
H	11.17232981	-4.82262702	0.16957161
H	10.58038278	-4.05394205	1.64683197
H	9.61341954	-5.29919675	0.83811381
C	10.49483604	-2.18450693	-0.48249128
H	10.80376198	-1.67605686	0.42725767
H	11.38916785	-2.51477860	-1.01406902
H	9.97289680	-1.47255279	-1.12452450
