# Supplementary material for: Efficient Electron Transfer Driven by Excited-State Structural Relaxation in Corrole–Perylenedimiide Dyad
Source: J Phys Chem Lett. 2024 May 8;15(19):5231–8. doi: 10.1021/acs.jpclett.4c00916 (PMC11103693; doi:10.1021/acs.jpclett.4c00916)
Supplement: Supplementary file 3 — jz4c00916_si_003.pdf [file jz4c00916_si_003.pdf]

jz-2024-00916a.R1

Name: Peer Review Information for "Efficient Electron Transfer Driven by Excited State Structural Relaxation in Corrole-Perylenedimiide Dyad"

First Round of Reviewer Comments

Reviewer: 1

Comments to the Author

This is a nice manuscript and should be published in JPCL because it offers interesting insights in the photo physics of corroles. Only a few additions are to be done:

- The dual fluorescence reported in Figure 2 is remarkable because of indication of a partial energy transfer. As a consequence, fluorescence excitation spectra should be recorded from 400 nm along the whole region of absorption both with monitoring 600, 700 and 750 nm.

- The molar absorptivities of the new dyes should be reported (such as in the SI).

- The influence of densely packing of PDI on the UV/Vis spectra was recently reported (https://doi.org/10.1021/acs.joc.3c01694.); the paper should be briefly considered and cited.

- The synthesis of PDI-Br was firstly described in https://doi.org/10.1002/(SICI)1099-0690(200001)2000:2<365::AID-EJOC365>3.0.CO;2-R. The paper should be cited in the SI.

Reviewer: 2

Comments to the Author

This article, by Prof. D. Gryko and coll., describes the synthesis and full spectroscopic characterization of a weakly coupled corrole-perylene dyad as well as the very efficient electron transfer between the donor and the acceptor thanks to an excited state dihedral motion that is quite remarkable.

The present article nicely shows the key importance of interchromophoric torsion motion in excited state and thus fully deserves publication in J. Phys. Chem. Lett.

Minor corrections or suggestions are noted below:

1) Figure 1 and TOC. The way you draw Cor-Ph-PDI makes it difficult for the reader to see the structure. I suggest drawing it as shown in Figure S10 (looks better to me).

2) Supporting information. NMR Data. When possible, the coupling constant values should be reported with the notation xJAB where the superscript x represents the number of bonds between the coupling atoms A and B (subscript).

3) Experimental section: give the yields, the weight but also the number of moles for all the compounds (e.g. PDI-PhCHO, BiPh-CHO, Cor-Ph-PDI…).

4) Use the same notation all over the ms for Cor-Ph-PDI (written PDI-corrole in the experimental section).

5) Supporting information. Check Figure S2. It looks like there is a problem with the position of the vertical bars.

6) Supporting information. Figures S6 and S7. Add the peak potential values on the CVs (oxidation and reduction)

7) Supporting information. NMR Spectra. Remove the Y axis on the right side. In a few cases, change the peak picking threshold as too many peaks have been indexed (see e.g Figures S10, S11).

8) Supporting information. MS and HRMS. If available, add the MS and HRMS spectra. Add also the Chemical Structures with the expected MS value and the delta in ppm between the experimental and calculated peaks.

9) References. Fix Ref #57 (A2B-corroles) and Ref#58 (subscripts and superscripts in the chemical formula)

Author's Response to Peer Review Comments:

Professor Daniel Gryko
Director

+48 22 343 23 21
daniel.gryko@icho.edu.pl

Senior Editor
The Journal of Physical Chemistry Letters

Warsaw 24 April 2024.

Dear Editor

We thank you very much for evaluating our manuscript entitled "Efficient Electron Transfer Driven by Excited State Structural Relaxation in Corrole-Perylenedimiide Dyad" (jz-2024-00916a) for possible publication in *The Journal of Physical Chemistry Letters*. We thank the reviewers for agreeing to review our work, and very much appreciate their positive remarks regarding our scientific work. The constructive comments and feedbacks by the reviewers have greatly helped us to improve the quality of our manuscript, and we thank all of them very much.
We have carefully addressed them regarding their questions and suggestions, as detailed in the next pages, and have modified the manuscript and the supporting information accordingly to reflect the suggested improvements. All changes made in the manuscript and supporting information have been highlighted in this letter and in the manuscript and supporting information for a clear reference.
We sincerely hope that based on the revised version of our manuscript, you can accept our work for publication in *The Journal of Physical Chemistry Letters* and we remain fully available for further questions and requests from your side.
<u>Non-scientific issues:</u>

Comment 1. Please submit your publication files without any markups. Please include annotated version(s) of your revised publication file(s) with colored text or highlights indicating the revisions that you have made as "Supporting Information for Review Only."
Response: We submitted both the files without any markups and highlighted versions.

Comment 2. Abstract: Shorten the abstract to 150 words or fewer.
Response: Abstract has been shortened to 142 words.

Comment 3. Figures: Please ensure that all parts of Figure 2 are labeled.
Response: Figure 2B was corrected.

Comment 4. TOC Graphic: Please resize the TOC graphic per journal guidelines (2 in x 2 in) and move to the correct position (on the same page as the abstract).
Response: The graphical abstract has been formatted according to the journal's guidelines and placed on the first page

Comment 5. References: In both the main file and the supporting information, fix the style of all references to use JPCL formatting (check all references carefully). ***JPC Letters reference formatting requires that journal references should contain: () around numbers; author names; article title (titles entirely in title case or entirely in lower case); abbreviated journal title (italicized); year (bolded); volume (italicized); and pages (first-last). Book references should contain author names; book title (in the same pattern); publisher; city; and year. Websites must include date of access.
Response: The references in both the main file and the experimental section have been corrected according to the guidelines

Comment 6. Supporting Information Statement: The supporting-information (SI) paragraph in the main file is incomplete. Please list each item (including graphics) that can be found in the SI-for-Publication file individually.
*Examples of sufficient descriptions: "Supporting Information: 1H NMR spectra for all compounds" or "Additional experimental details, materials, and methods, including photographs of experimental setup".
*Examples of insufficient descriptions: "Supporting Information: Figures S1-S3" or "Additional figures as mentioned in the text".
Response: The supporting information statement in the main file was corrected as follows:
,,The Supporting Information is available free of charge on the ACS Publications website.
Details of the synthetic procedures, characterization details, ^{1}H, $^{13}C\{1H\}$ NMR and HRMS spectra, computational studies, photophysical measurements, electrochemistry, and additional figures as mentioned in the text. (PDF),,

Comment 7. Supporting Information: Please number SI pages in the following format: "S1, S2…"
Response: The page numbering in the SI has been introduced in accordance with the requirements.

Reviewer: 1

This is a nice manuscript and should be published in JPCL because it offers interesting insights in the photo physics of corroles. Only a few additions are to be done:
We thank the Reviewer for her/his positive opinion about our manuscript.
Comment 1. The dual fluorescence reported in Figure 2 is remarkable because of indication of a partial energy transfer. As a consequence, fluorescence excitation spectra should be recorded from 400 nm along the whole region of absorption both with monitoring 600, 700 and 750 nm.
Response: The fluorescence excitation spectra of Cor-Ph-PDI in toluene were added to SI with observation at 517, 557, 600, 653, 690 and 720 nm.

Comment 2. The molar absorptivities of the new dyes should be reported (such as in the SI).
Response: The molar absorption coefficients spectra of Cor-Ph-PDI and Cor-BiPh in toluene were added to SI.

Comment 3. The influence of densely packing of PDI on the UV/Vis spectra was recently reported (https://doi.org/10.1021/acs.joc.3c01694.); the paper should be briefly considered and cited.

Response: We have included an additional comment regarding the design of the final dyad and the role of the imide substituent in potential molecule aggregation. A reference to the reviewer suggested publication has also been added.

,,During the design of the final compound molecule **Cor-Ph-PDI**, we utilized a branched imide substituent $CH(C_6H_{13})_2$ (swallow-tail substituents), which not only enhances solubility but also prevents aggregation at high concentrations.[58] ,,

Comment 4. The synthesis of PDI-Br was firstly described in https://doi.org/10.1002/(SICI)1099-0690(200001)2000:2<365::AID-EJOC365>3.0.CO;2-R. The paper should be cited in the SI.
Response: An additional reference has been added in the supporting information

Reviewer: 2

This article, by Prof. D. Gryko and coll., describes the synthesis and full spectroscopic characterization of a weakly coupled corrole-perylene dyad as well as the very efficient electron transfer between the donor and the acceptor thanks to an excited state dihedral motion that is quite remarkable. The present article nicely shows the key importance of interchromophoric torsion motion in excited state and thus fully deserves publication in J. Phys. Chem. Lett.
We thank the Reviewer for her/his positive opinion about our manuscript.

Minor corrections or suggestions are noted below:
Comment 1. Figure 1 and TOC. The way you draw Cor-Ph-PDI makes it difficult for the reader to see the structure. I suggest drawing it as shown in Figure S10 (looks better to me).
Response: We replaced the structure of Cor-Ph-PDI with the one presented in Figure S10 (now Figure S12) both in Fig. 1 and TOC.

Comment 2. Supporting information. NMR Data. When possible, the coupling constant values should be reported with the notation xJAB where the superscript x represents the number of bonds between the coupling atoms A and B (subscript).
Response: The NMR data has been expanded following the reviewer's suggestions.

Comment 3. Experimental section: give the yields, the weight but also the number of moles for all the compounds (e.g. PDI-PhCHO, BiPh-CHO, Cor-Ph-PDI...).
Response: The synthetic procedures in the experimental section of the supporting information have been supplemented with the number of moles of obtained products.

Comment 4. Use the same notation all over the ms for Cor-Ph-PDI (written PDI-corrole in the experimental section).
Response: The nomenclature of compounds has been standardized in both the main section of the publication and in the supporting information.

Comment 5. Supporting information. Check Figure S2. It looks like there is a problem with the position of the vertical bars.
Response: A vertical line corresponding to the vertical excitation energy of each excited state was inserted in Figure S2. These correspond to the data shown in Table S1a of the ESI.

Comment 6. Supporting information. Figures S6 and S7. Add the peak potential values on the CVs (oxidation and reduction)
Response: Cyclic voltammograms have been supplemented with peak potential values.

Comment 7. Supporting information. NMR Spectra. Remove the Y axis on the right side. In a few cases, change the peak picking threshold as too many peaks have been indexed (see e.g Figures S10, S11).
Response: The NMR spectra figures have been updated in accordance with the reviewer's suggestions.

Comment 8. Supporting information. MS and HRMS. If available, add the MS and HRMS spectra. Add also the Chemical Structures with the expected MS value and the delta in ppm between the experimental and calculated peaks.
Response: The supporting information has been updated with MS spectra

Comment 9. References. Fix Ref #57 (A2B-corroles) and Ref#58 (subscripts and superscripts in the chemical formula)
Response: Superscripts in the chemical formulas in the publication titles have been corrected.

On behalf of all authors, yours sincerely,

Daniel T. Gryko
